# Genome-Wide Analysis of the Gene Structure, Expression and Protein Interactions of the Peach (*Prunus persica*) *TIFY* Gene Family

**DOI:** 10.3389/fpls.2022.792802

**Published:** 2022-02-17

**Authors:** Yu Sheng, Hong Yu, Haifa Pan, Keli Qiu, Qingmei Xie, Hongli Chen, Songling Fu, Jinyun Zhang, Hui Zhou

**Affiliations:** ^1^Key Laboratory of Genetic Improvement and Ecophysiology of Horticultural Crops, Institute of Horticulture, Anhui Academy of Agricultural Sciences, Hefei, China; ^2^School of Forestry and Landscape Architecture, Anhui Agricultural University, Hefei, China; ^3^School of Life Sciences, Anhui Agricultural University, Hefei, China

**Keywords:** *TIFY*, JAZ, jasmonate, peach, *Prunus persica*, MeJA, MYC2

## Abstract

The *TIFY* family is a plant-specific gene family involved in regulating many plant processes, such as development and growth, defense and stress responses, fertility and reproduction, and the biosynthesis of secondary metabolites. The v2.0 peach (*Prunus persica)* genome, which has an improved chromosome-scale assembly and contiguity, has recently been released, but a genome-wide investigation of the peach *TIFY* family is lacking. In this study, 16 *TIFY* family genes from the peach genome were identified according to the peach reference genome sequence information and further validated by cloning sequencing. The synteny, phylogenetics, location, structure, and conserved domains and motifs of these genes were analyzed, and finally, the peach *TIFY* family was characterized into 9 *JAZ*, 1 *TIFY*, 1 *PPD* and 5 *ZML* subfamily members. Expression profiles of peach *JAZ*, *PPD*, and *ZML* genes in various organs and fruit developmental stages were analyzed, and they showed limited effects with fruit ripening cues. Four *TIFY* members were significantly affected at the mRNA level by exogenous treatment with MeJA in the peach epicarp, and among them, *PpJAZ1*, *PpJAZ4* and *PpJAZ5* were significantly correlated with fruit epicarp pigmentation. In addition, the TIFY family member protein interaction networks established by the yeast two-hybrid (Y2H) assay not only showed similar JAZ-MYC2 and JAZ homo- and heterodimer patterns as those found in Arabidopsis but also extended the JAZ dimer network to ZML-ZML and JAZ-ZML interactions. The PpJAZ3-PpZML4 interaction found in this study suggests the potential formation of the ZML-JAZ-MYC complex in the JA-signaling pathway, which may extend our knowledge of this gene family’s functions in diverse biological processes.

## Introduction

The *TIFY* family, previously known as *ZIM* (zinc finger protein expressed in the inflorescence meristem), is a plant-specific gene family involved in regulating many plant processes, such as development and growth, defense and stress responses, fertility and reproduction, and the biosynthesis of secondary metabolites. The *TIFY* gene family is divided into four phylogenetic subfamilies, *TIFY* (threonine, isoleucine, phenylalanine, tyrosine), JAZ (jasmonate-ZIM-domain), *PPD* (PEAPOD), and *ZML* (zinc finger protein expressed in inflorescence meristem, TIFY-like). All four subfamilies contain a conserved TIFY domain (Nishii et al., 2000). In addition to this domain, ZMLs have CCT and GATA Zn finger domains, and JAZ and PPD contain Jas [an S-L-X(2)-F-X(2)-K-R-X(2)-R core, delimited by a conserved N-terminal Pro and a C-terminal PY sequence] and modified Jas (lacking the conserved PY amino acids at the C-terminus) motifs, respectively (Yan et al., 2007; Staswick, 2008; Chung et al., 2009). However, TIFY subfamily members do not have these domains or motifs in addition to the TIFY domain (Staswick, 2008). Among these subfamilies, JAZ proteins are best characterized as key regulators of the jasmonic acid (JA) signaling pathway.

Jasmonates (JAs) and their derivatives are essential volatile compound phytohormones that play a key role in regulating defense responses and coordinating growth and development processes in plants (Howe et al., 2018). The broad functions of JAs indicate the flexibility of this hormone as a regulator of diverse biological processes. The JA signaling pathway is initiated with the perception of bioactive JA jasmonoyl-isoleucine (JA-Ile). The presence of JA-Ile leads to degradation of JAZ proteins by the ubiquitin-26S proteasome system, leading to the release of MYC (bHLH) transcription factors (TFs), which are key master regulators of JA responses (Chini et al., 2007; Thines et al., 2007; Browse, 2009). In addition, according to a recent report, JAZs mediate repressive chromatin modifications at JA-responsive genes by interacting with chromatin-associated polycomb proteins (Li et al., 2021).

Due to the important roles of the *TIFY* family (especially the JAZ subfamily) in diverse biological processes, this gene family has provoked great interest. The *TIFY* family has recently been investigated genome-wide, identified and analyzed in several plants, such as *Arabidopsis* (Vanholme et al., 2007), grape (Zhang et al., 2012), cotton (He et al., 2015; Sun et al., 2017), apple (Li et al., 2015), maize (Han and Luthe, 2021; Heidari et al., 2021; Sun et al., 2021), pigeonpea (Sirhindi et al., 2016), tomato (Chini et al., 2017; Heidari et al., 2021), Populus (Wang et al., 2017; Xia et al., 2017), wheat (Ebel et al., 2018; Singh and Mukhopadhyay, 2021), and pear (Ma et al., 2018). The *TIFY*, *JAZ*, *PPD*, and *ZML* members are divergent in gene number as a result of segmental and tandem duplications but retain the characteristic gene structures (domains and motifs) in seed plants. The functions of the conserved TIFY and Jas domains in JAZ subfamilies have been discovered in model plants such as *Arabidopsis* and tomato. The Jas domain plays an important role in the interaction between JAZs and CORONATINE-INSENSITIVE1 (COI1), the F-box subunit of the SCF*^COI^*^1^ ubiquitin ligase or MYC2 TFs, whereas the TIFY domain is involved in the recruitment of the corepressor NINJA. Some JAZs, such as AtJAZ7/8, contain the EAR motif, which mediates the interaction of JAZs and the TOPLESS (TPL) corepressor (Garrido-Bigotes et al., 2019). TPL also physically interacts with NINJA (Pauwels et al., 2010). When there are no bioactive JAs, JAZs bind with MYC2 *via* the Jas domain and recruit the repressor complex NINJA-TPL *via* the TIFY domain to repress JA-responsive genes. In contrast, in the presence of the bioactive JA ligand JA-Ile, JAZ proteins form a JA-Ile-dependent complex with COI1 and are subsequently degraded by the 26S proteasome, which leads to the release of MYC2 from JAZ-mediated repression. Moreover, the TIFY domain mediates the interactions between JAZ proteins to form homo- or heterodimers (Vanholme et al., 2007; Chini et al., 2009).

JAZ homo- or heterodimers have been reported in several plant species, such as *Arabidopsis* (Chini et al., 2009), *Hevea brasiliensis* (Hong et al., 2015), cotton (Li et al., 2017), and *Mentha canadensis* (Xu et al., 2021). A previous study suggested that dimerization may contribute to the stability of JAZ proteins or facilitate the simultaneous interaction of multiple JAZs with MYC proteins (Chini et al., 2009; Chung and Howe, 2009; Geerinck et al., 2010; He et al., 2020). Since the interactions between JAZ proteins are mediated by the TIFY domain and this domain also exists in the PPD and ZML subfamilies, the putative hetero-interactions of these members with JAZ subfamily members may also play important roles in diverse biological processes. However, this kind of hetero-interaction has seldom been reported before.

Several peach JAZ subfamily members have been investigated in previous studies. The expression levels of *PpJAZ2*, *PpJAZ7*, and *PpJAZ10* during the fruit developmental stages were found to be affected by exogenous treatment with 0.80 mM methyl jasmonate (MeJA) (Ruiz et al., 2013). In a later study, another JAZ member, *PpJAZ1*, was found to mediate the transition from outcrossing to self-pollination in peach (Sherif et al., 2015). In addition to *PpJAZ1*, another 8 putative JAZs were found according to phylogenetic analysis based on peach genome v1.0 (Sherif et al., 2015). However, due to the relatively low quality of the gene annotation of the peach genome version 1.0 and no analysis of domains or motifs provided, fine analysis needs to be addressed to further characterize the peach *TIFY* family, including *JAZ*s. Here, we report a genome-wide analysis of the peach TIFY family. The gene structures, collinearity relationships, gene expression patterns and protein interaction networks of the JAZ, PPD and ZML subfamilies were analyzed in this study, and the results provide valuable information on this gene family and promote research on development and growth, defense and stress responses, fertility and reproduction, as well as the biosynthesis of secondary metabolites in which this gene family is involved.

## Materials and Methods

### Data Collection

To identify candidate *TIFY* family members in the peach genome, Hidden Markov model (HMM) profiles of the TIFY domain (PF06200) and Jas (CCT-2) domain (PF09425) and the CCT domain (PF06203) were used as queries to search the peach *TIFY* members in the peach genome v2.0 (Verde et al., 2013, Verde et al., 2017) using hmmsearch (HMMER, v3.2.1) software (Finn et al., 2011). The *E*-value threshold of the full-length sequence alignment was set as e-05, and only the protein accessions with E-values lower than the threshold were listed as candidate hits.

### Gene Structure, Collinearity, and Phylogenetic Analysis

Candidate domains and motifs were verified by SMART^1^ and NCBI CDD^2^ databases with default parameters. Collinearity analysis within the peach genome and synteny analysis between the peach genome v2.0^3^ and apple genome v1.0, which was downloaded from Phytozome database^4^, were conducted using MCScanX software (Wang et al., 2012). The phylogenetic analysis was conducted using MEGA software (v10.0.5). The full-length deduced amino acid sequences were aligned using Muscle software. The phylogenetic tree was constructed using the maximum likelihood method with a bootstrap test of 1,000 replicates. Gaps were treated with the partial deletion option with site coverage cutoff set at 70% to keep as much potentially phylogenetical information as possible. The final tree was visualized using Interactive Tree of Life (ITOL^5^). The following GenBank or genome sequencing project accession numbers were used: *Arabidopsis thaliana* AtJAZ1 (At1g19180), AtJAZ2 (At1g74950), AtJAZ3 (At3g17860), AtJAZ4 (At1g48500), AtJAZ5 (At1g17380), AtJAZ6 (At1g72450), AtJAZ7 (At2g34600), AtJAZ8 (At1g30135), AtJAZ9 (At1g70700), AtJAZ10 (At5g13220), AtJAZ11 (At3g43440), AtJA Z12 (At5g20900), AtZML1 (At3g21175), AtZML2 (At1g51600), AtPPD1 (AT4g14713), and AtPPD2 (AT4G14720); *Malus* × *domestica* MdJAZ1 (MDP0000187921), MdJAZ2 (MDP00003 01927), MdJAZ3 (MDP0000193833), MdJAZ4 (MDP00001353 75), MdJAZ5 (MDP0000174042), MdJAZ6 (MDP0000718271), MdJAZ7 (MDP0000173534), MdJAZ8 (MDP0000173535), MdJA Z9 (MDP0000889413), MdJAZ10 (MDP0000565690), MdJAZ11 (MDP0000891920), MdJAZ12 (MDP0000452772), MdJAZ13 (M DP0000244580), MdJAZ14 (MDP0000243322), MdJAZ15 (MDP 0000871409), MdJAZ16 (MDP0000285658), MdJAZ17 (MDP000 0241358), MdJAZ18 (MDP0000757701), MdPPD1 (MDP000025 7732), MdPPD2 (MDP0000282472), MdZML1 (MDP00005098 77), and MdZML2 (MDP0000159765); *Vitis vinifera* VvJAZ1 (XM_002284819), VvJAZ2 (XM_002262714), VvJAZ3 (XM_0036 34778), VvJAZ4 (XM_002272327), VvJAZ5 (XM_002277733), VvJAZ6 (XM_002277769), VvJAZ7 (XM_002277916), VvJAZ8 (CBI30922), VvJAZ9 (XM_002277121), VvJAZ10 (XM_002263 220), VvJAZ11 (XM_002282652), VvPPD1 (XM_002279284), VvPPD2 (CBI25038), VvZML1 (XM_002270325), VvZML2 (XM_002263671), VvZML3 (XM_002283717) and VvZML4 (XM_002283702); *Oryza sativa* OsJAZ1 (Os04g55920), OsJA Z2 (Os07g05830), OsJAZ3 (Os08g33160), OsJAZ4 (Os09g2 3660), OsJAZ5 (Os04g32480), OsJAZ6 (Os03g28940), OsJAZ7 (Os07g42370), OsJAZ8 (Os09g26780), OsJAZ9 (Os03g08310), OsJAZ10 (Os03g08330), OsJAZ11 (Os03g08320), OsJAZ12 (Os10g25290), OsJAZ13 (Os10g25230), OsJAZ14 (Os10g252 50), and OsJAZ15 (Os03g27900); *Physcomitrella patens* JAZs (PP00103G00080, PP00442G00070, Pp3c5_11800, Pp3 c6_23650, Pp3c25_6330, Pp3c16_13490, Pp3c5_11730 and Pp3 c25_6300), PpaZML1 (gw1.68.123.1), PpaZML2 (fgenesh 1_pm.scaffold_69000012), PpaZML3 (fgenesh2_pm.scaffold_11 1000001), and PpaZML4 (e_gw1.226.30.1).

### Plant Materials

Eight-year-old “Maravilha” and “124 Pan” peach trees are maintained at Wuhan Botanical Garden of the Chinese Academy of Sciences (Wuhan, China). Seven-year-old “Huang Jin Mi,” “Zhong You 18,” and “Qing Feng” peach trees are maintained at Institute of Horticulture, Anhui Academy of Agricultural Sciences (Hefei, China). Fruit samples of “Maravilha” and “124 Pan” were collected at the following developmental stages: S1 (the first exponential growth), S2 (the pit hardening), S3 (the second exponential growth), and S4 (fruit ripening). Fruit samples of “Huang Jin Mi,” “Zhong You 18,” and “Qing Feng” were collected at stage S3. Each fruit sample consisted of three biological replicates from three different trees of the same cultivar, and each biological replicate contained at least five fruits collected from one tree. Fruits were cored, cut into pieces, immediately frozen in liquid nitrogen for at least 5 min and then stored at -80°C until use.

### RNA-Seq Data Analysis and Heatmap Construction

The raw RNA-Seq data were downloaded from the Sequence Read Archive (SRA)^6^ database, with peach transcriptome data of flowers (PRJNA726283, three biological replicates of petal tissues at blooming stage of a 3-year-old F_2_ individual generated from self-pollination of a peach accession “05-2-144,” Lu et al., 2021), roots and leaves (PRJEB12334, three biological replicates of root samples and leaf tissues from 15 1-year-old “GF277” peach trees and 15 1-year-old “Catherina” peach trees, respectively, Ksouri et al., 2016), shoots (PRJNA587386, three biological replicates from three different 5-year-old “Soomee” peach trees with three shoots each was used, Yu et al., 2020) and fruits (PRJNA576753, three biological replicates of fruit samples at stages S1, S2, S3, S4, and S5 of cv. “Hujingmilu” with five fruits each were used at each sample time, Cao et al., 2019). Adaptors and low-quality read filtering were conducted using fastp software v0.20.1 (Chen et al., 2018). Then, the clean reads were mapped into the v2.0 peach genome using hisat2 (Kim et al., 2015) version 2.2.1 with default parameters. Quantification of the gene expression levels of the genes was conducted using the R package Rsubread v2.4.3 (Liao et al., 2019). The gene expression levels were quantified using transcripts per kilobase million (TPM). A heatmap of the gene expression levels in various tissues and fruit developmental stages was constructed using TBtools software v1.082 (Chen et al., 2020).

### Exogenous Methyl Jasmonate Treatment of the Peach Fruits

According to the previous reports, the minimum concentration of MeJA used for fruits treatment is usually 50 μM and the maximum concentration is usually lower than 1000 uM (Ju et al., 2016; Wei et al., 2017), therefore in this study, fruits were immersed in solutions with different concentrations of MeJA (50, 200, and 1000 μM) [containing 0.01% dimethylsulfoxide (DMSO)] or distilled water (containing 0.01% DMSO) for 10 min and then transferred to plant growth chambers at 25°C under a light/dark cycle of 16/8 h. Fruit epicarp samples were collected at 2, 4, and 6 days after MeJA treatment.

### Measurement of Anthocyanin Content

Measurement of anthocyanin content was conducted according to a previous report (Zhou et al., 2014) with some modifications. Briefly, approximately 0.5 g of epicarp tissue was ground to fine powder in liquid nitrogen, and the anthocyanins were extracted with 15 ml extraction solution (1% HCl in 70% ethanol) for 24 h. After centrifugation, 1 ml supernatant was mixed with 4 ml buffer A (0.2M KCl: 0.2M HCl = 25:67, pH = 1.0) or buffer B (1M sodium acetate: 1M HCl: H2O = 50:30:45, pH 4.5). Absorbance of each mixture was measured at 510 and 700 nm. The anthocyanin content was calculated using the following equation: TA (mg/100 g) = A*MW*75*5*100/(26900*0.5).

### RT–qPCR

Total RNA extraction was conducted using a Total RNA Rapid Extraction Kit (Zomanbio, Beijing, China). First strand cDNA synthesis was performed using the PrimeScript™ RT Reagent Kit with gDNA Eraser (Takara Bio, Inc.). RT–qPCR was conducted using TB Green^®^ Premix Ex Taq™ (Tli RNaseH Plus, Takara Bio, Inc.), with the following program: one cycle of 30 s at 95°C, followed by 40 cycles of 5 s at 95°C and 30 s at 60°C. The previously reported translation elongation factor gene *PpTEF2* was used as the internal reference gene (Tong et al., 2009). Three biological replicates were conducted for each sample. Sequences of the primers used for RT–qPCR are listed in Supplementary Table 1.

### Yeast Two Hybrid Assay

The full-length coding sequences of peach JAZs, ZMLs and MYC2 were amplified using a list of primers (Supplementary Table 1). PCR products were digested with restriction enzymes and inserted into the Y2H vectors *pGBKT7* and *pGADT7* as bait and prey, respectively. The yeast two-hybrid assay was conducted using the Matchmaker^®^ Gold Yeast Two-Hybrid System (Clontech, Japan). The bait and prey vectors (empty vectors as negative controls) were transformed into yeast strains “Y2Hgold” and “Y187” using the Frozen-EZ Yeast Transformation II Kit (Zymo RESEARCH), respectively. After mating, the diploid yeast cells were grown on DDO (SD-Trp-Leu) and QDO/A/X (SD-Trp-Leu-Ade-His + AbA + X-α-Gal) media at 30°C. Photos were taken after 3 days following incubation.

### Split Firefly Luciferase Complementation Assay

Split firefly luciferase complementation assays were conducted on the young *Nicotiana benthamiana* leaves according to a previous report (Chen et al., 2008). Briefly, whole-coding sequences of *PpJAZ3* and *PpZML4* (with the native stop codon removed) were both amplified and separately inserted into multiple cloning site (MCS) of the binary vector *pCambia1300NLuc*, while the whole coding region of *PpMYC2* (without ATG translation initiation codon) was cloned and inserted into MCS of the binary vector *pCambia1300CLuc*. These recombinant constructs and the empty vectors were individually transferred into *Agrobacterium* strain GV3101 *via* electroporation and incubated at 28°C for 2 days. The confluent bacteria were suspended in the buffer containing 10 mM MES, 10 mM MgCl_2_ and 200 μM acetosyringone and incubated at room temperature for 2 h. Agrobacterium cultures containing the NLuc and CLuc derivatives were mixed in equal ratio and injected into young leaves of *Nicotiana benthamiana* seedlings grown in the greenhouse using needleless syringes. At the 3rd days after infiltration, the infiltrated leaves were soaked with one millimolar D-luciferin, sodium salt (Yeasen, Shanghai, China) in dark for 6 min to quench the fluorescence. The Luc images were captured using a cooled CCD imaging apparatus (Tanon 5200 Multi-Imaging System, Tanon Science and Technology Inc., Shanghai, China). Leaf discs (1 cm in diameter) adjacent to the infiltration holes were punched to measure firefly luciferase (Luc) activities using Bright-Glo Luciferase Assay System (Promega) on an Infinite M200 luminometer (Tecan, Mannerdorf, Switzerland).

## Results

### Genome-Wide Identification and Cloning of the *TIFY* Gene Family in the Peach Genome

Hidden Markov model profiles of the TIFY, the Jas (CCT-2) and the CCT domains were used as queries to screen the peach genome (v2.0) to identify the putative peach *TIFY*, *JAZ*, *PPD* and *ZML* subfamily members. In total, 15, 16 and 28 protein hits were found to possess the TIFY domain, the Jas domain and the CCT motif, respectively, with 15 of them containing both the TIFY domain and the Jas domain and 5 of them comprising both the TIFY domain and the CCT motif (Supplementary Table 2). Prupe.1G467500 contains a TIFY domain but not a Jas or CCT motif and was therefore assigned as a member of the TIFY subfamily. Considering the limited functional study and information about the TIFY subfamily, this study focused on TIFY family members containing the Jas or CCT motif, and only one TIFY subfamily member, *Prupe.1G467500*, was not investigated further. It was interesting that all 5 members comprising both the TIFY domain and the CCT motif were included among the 15 members containing both the TIFY domain and the Jas domain, which meant that 5 members (Prupe.8G071200, Prupe.8G071300, Prupe.1G534400, Prupe.3G019800 and Prupe.1G534300) contained both the Jas domain and the CCT motif. To confirm these results and further classify the proteins, the SMART and NCBI CCD databases were used to examine the conserved domains of the 15 candidate proteins. Indeed, scanning of the SMART database showed the same results as the HMM software: the five proteins contained both the Jas domain and the CCT motif, and the two motifs overlapped with each other; however, the CCT motif showed much smaller E-values than the Jas domain for all five members (Supplementary Table 3). Furthermore, the NCBI CCD database called only the CCT motif but not the Jas domain for the five proteins. Therefore, five proteins (Prupe.8G071200, Prupe.8G071300, Prupe.1G534400, Prupe.3G019800 and Prupe.1G534300) were concluded to contain both the TIFY and CCT motifs and were predicted to belong to the ZML subfamily.

To further separate the JAZ and PPD subfamilies, amino acid alignment of all JAZs of peach and *Arabidopsis* was performed (Supplementary Figure 1) and the annotation of the domains was conducted according to a recent report (Garrido-Bigotes et al., 2020). One member, Prupe.1G020900, lacks the conserved PY motif at its C-terminal region, although it contains the TIFY domain and the Jas domain, which is consistent with the features of PPD proteins (Chini et al., 2009).

Taken together, 1 *TIFY*, 9 *JAZ*s, 1 *PPD* and 5 *ZML* genes were finally obtained in this study. Compared to a previous report of peach JAZs involved in fruit and seed development (Ruiz et al., 2013), 2 more *JAZ*s (*Prupe.7G189200* and *Prupe.1G578500*) were found in this study. Finally, we designated these JAZs following the rules made by Ruiz et al. (2013), with some supplements and modifications (Table 1). *Prupe.7G189200* and *Prupe.1G578500* were designated *PpJAZ2* and *PpJAZ6*, respectively. In addition, *Prupe.1G020900*, previously named *PpJAZ9*, was corrected to the name *PpPPD1*. To further confirm the structures of these genes, we cloned the genes using fruit cDNAs as templates and verified the gene structures by Sanger sequencing. Finally, 13 full-length CDSs of the 15 genes were successfully cloned, except *PpPPD1* and *PpJAZ6*, possibly due to their low expression levels in fruit. Most of the gene structures were the same as the genome sequencing annotation v2.0; however, some of them were not. For example, *PpJAZ3* was identical to the CDS of peach genome annotation v1.0 (*ppa008065m*) but not v2.0 (*Prupe.5G235300*). In addition, *PpJAZ8* (318 bp) was shorter than the CDS (*Prupe.3G188200*, 363 bp in length) in the v2.0 annotation. Similarly, compared to the gene structure of *Prupe.3G019800* annotated in the v2.0 peach genome, *PpZML4* skips one intron, which produces an internal stop codon and results in premature termination of translation. Nevertheless, the deduced protein of *PpZML4* still contains the TIFY domain and the CCT motif.

**TABLE 1:** The peach *TIFY* gene family.

Gene name	Accession number in this study	Amino acid length	Location	Gene name in previous reports (Ruiz et al., 2013; Sherif et al., 2015)
JAZ1	Prupe.7G194800	278	Pp07:18485565-18487499	JAZ1
JAZ2	Prupe.7G189200	216	Pp07:18154542-18156340	
JAZ3	ppa008065m	347	Pp05:17832236-17835446	JAZ3
JAZ4	Prupe.1G331500	377	Pp01:31407966-31410949	JAZ4
JAZ5	Prupe.3G037800	213	Pp03:2732646-2733608	JAZ5
JAZ6	Prupe.1G578500	273	Pp01:47075905-47078672	
JAZ7	Prupe.4G082500	124	Pp04:4029250-4030091	JAZ7
JAZ8	OK086788	105	Pp03:20163873-20164702	JAZ8
JAZ10	Prupe.1G218500	269	Pp01:23338321-23339881	JAZ10
PPD1	Prupe.1G020900	392	Pp01:1464406-1468733	JAZ9
ZML1	Prupe.8G071200	294	Pp08:10425086-10429487	
ZML2	Prupe.8G071300	366	Pp08:10433030-10440040	
ZML3	Prupe.1G534400	354	Pp01:43682892-43687775	
ZML4	OK086789	301	Pp03:1457573-1461524	
ZML5	Prupe.1G534300	296	Pp01:43675463-43678548	

*All the accession numbers with the prefixes “ppa,” “Prupe,” and “OK” were derived from peach genome sequence database v1.0, v2.0, and NCBI nucleotide database, respectively.*

### Expansion Patterns of the Peach *JAZ*, *PPD*, and *ZML* Subfamilies

**FIGURE 1:**
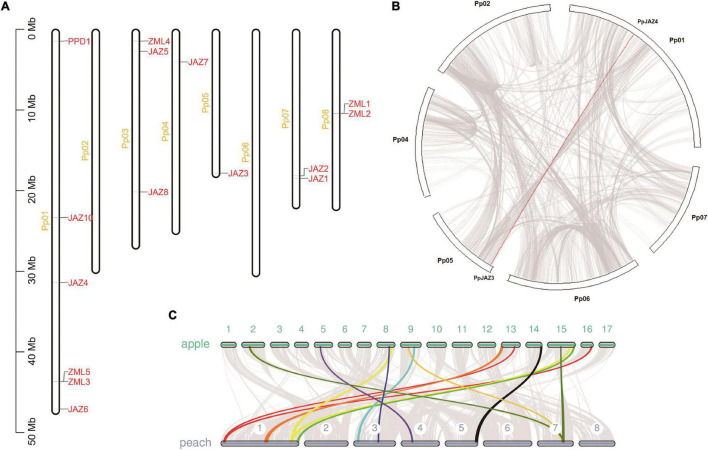
Genomic analysis of peach *JAZ*, *PPD* and *ZML* genes. **(A)** Genomic distribution of the *JAZ*, *PPD* and *ZML* genes in the peach genome v2.0 (Verde et al., 2017). The white bars represent peach chromosomes; **(B)** Collinearity analysis of the *JAZ*, *PPD*, and *ZML* members in the peach genome v2.0. The red line represents the segmentally duplicated pair, *PpJAZ3*/*4*; **(C)** Large-scale synteny comparison between peach genome v2.0 and apple genome v1.0 (Velasco et al., 2010). Colored bands represent the synteny regions comprising the peach *JAZ*, *PPD* or *ZML* genes.

The generation and maintenance of gene families is usually associated with tandem and segmental gene duplication. According to a previous report, tandem and segmental gene duplication play different roles in different gene families (Cannon et al., 2004). The peach *JAZ*, *PPD*, and *ZML* members were mapped to chromosomes 1, 3, 4, 5, 7, and 8 (Figure 1A and Table 1). Based on the positions of these genes and their phylogenetic relationships, we identified two ZML tandem repeat clusters (*PpZML1*/*2* and *PpZML3*/*5*) located on chromosomes 8 and 1, respectively. Collinearity analysis of the *JAZ*, *PPD*, and *ZML* members found only one segmentally duplicated pair, *PpJAZ3*/*4* (Figure 1B), which was located on chromosomes 5 and 1, respectively. These results revealed that 2 of the 9 *JAZ* and 4 of the 5 *ZML* genes were associated with either tandem or segmental gene duplication events.

### Evolutionary Relationships of the Peach and Apple *JAZ*, *PPD*, and *ZML* Genes

Peach and apple share a hypothetical ancestral *Rosaceae* genome with 9 chromosomes (Illa et al., 2011). Comparison of the *JAZ*, *PPD* and *ZML* genes between peach and apple can provide insights into the evolutionary history of this gene family. A previous report characterized 18 *JAZ*s, 2 *PPD*s and 2 *ZML*s in the apple genome (Li et al., 2015). A comparative syntenic analysis was conducted between peach and apple genomes. The *JAZ*s, *PPD*s and *ZML*s located in/around one identified large-scale synteny are highlighted in Figure 1C. The final syntenies contained homologs from peach and apple, including 7 peach *JAZ*s, 1 peach *PPD*, 1 peach *ZML* and 11 apple *JAZ*s, 2 apple *PPD*s, and 2 apple *ZML*s (Supplementary Table 4). One pair of orthologs, *PpJAZ8*-*MdTIFY2*, appeared to correspond to a single peach-to-apple JAZ gene. Considering the duplication event in the apple genome, we hypothesized that gene loss events occurred for these genes in the apple genome after whole genome duplication. There are also more instances where a single peach gene corresponded to multiple apple genes, including *PpJAZ4*-*MdJAZ12*/*18* (*MdJAZ18* was unanchored), *PpJAZ2*-*MdJAZ*2/9, *PpPPD1*-*MdPPD*1/2 and *PpZML3*-two unidentified apple *ZML*s (*MDP0000192617* and *MDP0000316985*). Most of these orthologs followed the whole genome duplication pattern. For example, *PpPPD1* is located on the proximal part of chromosome 1 of the peach genome, and *MdPPD1* and *MdPP2* are located on chromosomes 13 and 16 of the apple genome, which were both derived from the hypothetical ancestral *Rosaceae* chromosome A1 (Illa et al., 2011). Similarly, the two apple chromosomes 8 and 15, containing the two apple *ZML*s *MDP0000192617* and *MDP0000316985*, respectively, also shared the same hypothetical ancestral Rosaceae chromosome A2, with the distal part of peach chromosome 1, where *PpZML3* was located. Interestingly, duplication of *JAZ*s (*MdJAZ2* and *MdJAZ9*) on non-homologous chromosomes 2 and 9 of the apple genome, which are orthologs of *PpJAZ2*, was also observed in our analysis (Figure 1C and Supplementary Table 4).

The peach genomic region containing the *ZML* tandem repeat *PpZML1*/*PpZML2* is homologous with regions on chromosomes 5 and 10 of the apple genome; however, there were no *ZML* genes in this apple genomic region. For another *ZML* tandem repeat, *PpZML3*/*PpZML5*, only *PpZML3* was in a synteny with two apple *ZML*s, which implied that *PpZML5 was* probably generated from *PpZML3* by tandem duplication.

### Phylogenetic Analysis of the *JAZ*, *PPD*, and *ZML* Genes of Different Plant Species

**FIGURE 2:**
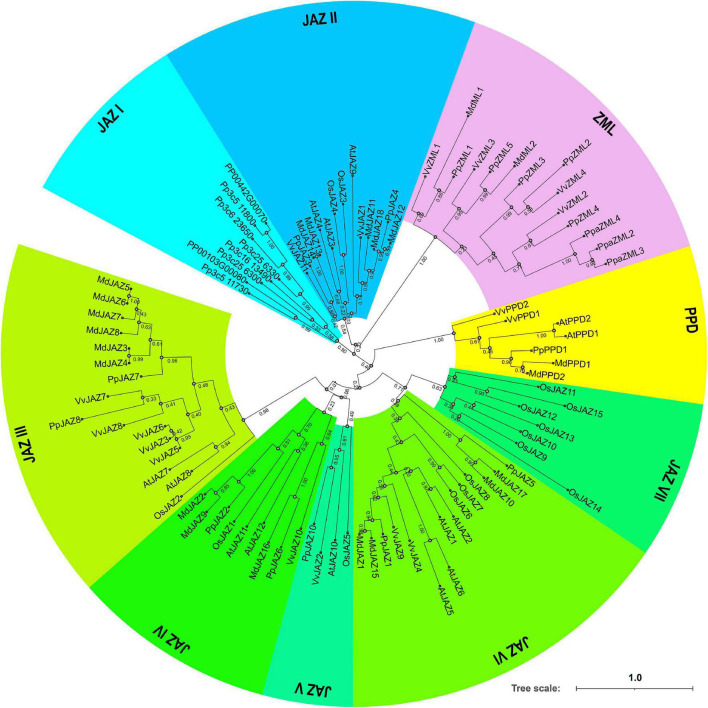
A phylogenetic tree derived from deduced amino acid sequences of *JAZs*, *PPDs* or *ZMLs* in moss (Ppa, *Physcomitrella patens*), peach (Pp, *Prunus persica*), apple (Md, *Malus* × *domestica*), grape (Vv, *Vitis vinifera*), rice (Os, *Oryza sativa*), and Arabidopsis (At, *Arabidopsis thaliana*). Sequences were aligned using MUSCLE software (Supplementary Data Sheet 1), and the phylogenetic tree was constructed using the maximum-likelihood method with MEGA-X. The tree is drawn to scale and the scale bar represents 1.0 substitutions per site. Values under each node represent bootstrap values. All the gene accession numbers were list in Section “Materials and Methods”.

To further understand the evolutionary relationship of the JAZ, PPD and ZML genes in different species, a phylogenetic tree was constructed using the maximum likelihood method (Figure 2). The deduced whole-length protein sequences of JAZ, PPD and ZML proteins from the moss *Physcomitrella patens* (Bai et al., 2011), Arabidopsis (Chini et al., 2009), rice (Ye et al., 2009), grape (Zhang et al., 2012), apple (Li et al., 2015) and peach were extracted and used in this analysis. The phylogenetic analysis results showed that all of the JAZ, PPD and ZML members were classified into 9 clades, comprising 1 PPD clade, 1 ZML clade and 7 JAZ clades. The moss ZMLs were clustered with ZMLs from the other species and grouped into the ZML clade; however, moss JAZs clustered into one clade (JAZ I), distinguishing them from JAZs from eudicots, including peach (Figure 2). These results indicated that JAZs divergent at a faster rate than ZMLs during evolution history. Moreover, similar to a previous report (Zhang et al., 2012), PPD clade members were not present in *P. patens* but in all dicot plants, indicating the possible late emergence of the PPD domain following the divergence of moss and vascular plants.

When comparing the phylogenetic relationships of these genes between peach and apple, it was found that most of the peach JAZ, PPD and ZML members showed the highest homology with apple genes due to their close evolutionary relationship. For most of the clades, including JAZ I, JAZ III, JAZ IV, JAZ V, and PPD, apple always contained more members than peach (Figure 2). This result is consistent with the genome duplication event that occurred during *Rosaceae* evolution. However, this rule did not work in the ZML clade, which was in accordance with the syntenic analysis results between peach and apple. These results suggested that gene duplication and loss events occurred during the evolution of *Rosaceae* species.

### Gene Structure Analysis of the Peach *JAZ*, *PPD*, and *ZML* Genes

**FIGURE 3:**
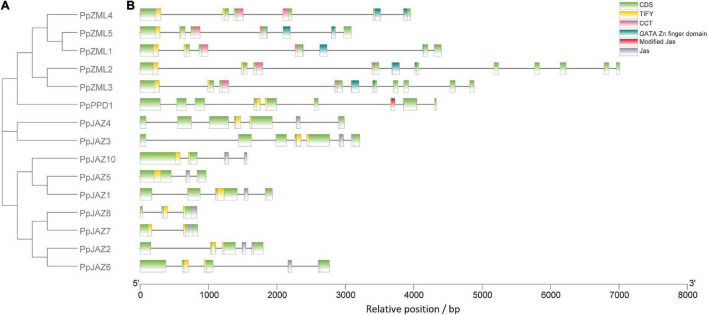
Structural analysis of the peach JAZ, PPD, and ZML members. **(A)** The topology of the bootstrap consensus tree inferred from deduced amino acid sequences of peach JAZ, PPD, and ZML members with 1000 replicates; **(B)** Locations of the exons, introns, domains and motifs for each peach member. The *x*-axis shows the relative position in the peach reference genome. Introns are represented by black lines, whereas exons and domains are represented by colored boxes, as shown in the upper right corner.

Phylogenetic analysis was also conducted using the deduced full-length protein sequences of the 15 peach JAZ, PPD and ZML genes identified in this study (Figure 3A). The resulting topological structure was consistent with the phylogenetic tree constructed from gene sequences from five different species (Figure 2), in which the JAZs and ZMLs clustered into two different clades.

The exon/intron structures were also investigated according to the location of the exon and intron sequences in the peach genome (Figure 3B). The results showed that the genes in the same phylogenetic clades tended to share the same or similar exon/intron structures. *PpJAZ2*/*6*, *PpJAZ3*/*4* and *PpZML1*/*5* had identical numbers of exons with similar lengths, which suggested that they may be the products of gene duplication. Interestingly, *PpZML2*/*3* also had similar exon-intron patterns. Considering that tandem duplication events occurred in *PpZML1*/*2* and *PpZML3*/*5*, it is reasonable to speculate that the orthologs (*PpZML1*/*5* or *PpZML2*/*3*) were generated later than the paralogs (*PpZML1*/*2* or *PpZML3*/*5*). Nevertheless, the exon length was not conserved for genes having the same or similar exon numbers and length. For example, the first intron of *PpJAZ3* (1.36 Kb in length) is much larger than that of *PpJAZ4* (0.46 Kb in length), and the same occurred for the fifth intron of *PpZML1* and *PpZML5*.

To further confirm the evolutionary relationships among the peach *JAZ*, *PPD* and *ZML* genes, the distribution of the conserved domains and motifs was assessed (Figure 3B). All members in the same clades shared the same conserved domains and motifs. For example, all JAZs have the TIFY domain and Jas domain, and all ZMLs contain the TIFY, CCT and GATA Zn finger domains. It is interesting that both JAZs and ZMLs have the TIFY domain, but ZMLs showed a more conserved location and distribution of this domain than JAZs. The TIFY domain of ZMLs always spans the first and second exons but does not always exhibit this pattern for JAZs. These results suggested that JAZs showed more divergent structures than ZMLs, probably implying more divergent functions of JAZs.

### Expression Profiles of Peach *JAZ*, *PPD*, and *ZML* Genes in Various Organs and Fruit Developmental Stages

**FIGURE 4:**
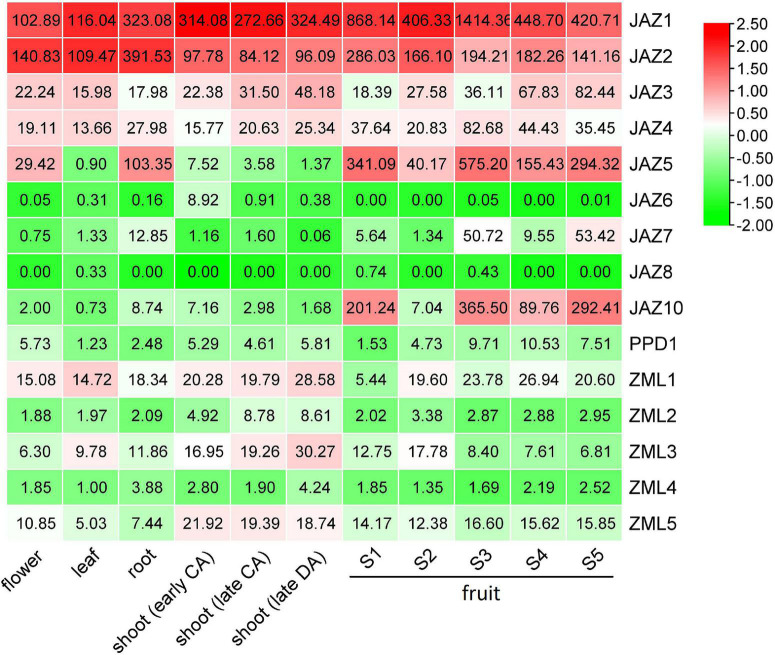
Heatmap analysis of tissue-specific expression patterns of peach *JAZ*, *PPD* and *ZML* genes. The original TPM values of genes were normalized with a log transform and mean center columns. The values given in the boxes represent the original TPM values of genes, whereas the indicator ranges in the upper right corner represent the range of the values after normalization. CA, cold acclimation; DA, deacclimation; shoots during early CA, late CA and early DA represent shoots at the end of October, middle of January and middle of March, respectively; S1, the first exponential growth stage; S2, the pit hardening stage; S3, the second exponential growth; S4, fruit ripening stage; S5, fruit post ripening stage.

To investigate the tissue-specific expression patterns of the *JAZ*, *PPD* and *ZML* genes of peach, RNA-Seq data was downloaded from NCBI SRA database, including peach transcriptome data of flowers, roots, leaves, shoots and fruits. A heatmap was constructed according to gene expression levels (TPMs) in various tissues (Figure 4 and Supplementary Table 5). The heatmap results showed that most of the genes in this gene family were expressed in a tissue-specific pattern. For example, the expression of *PpJAZ10* was much higher in the fruit than in the other tissues investigated. *PpJAZ5* showed lower mRNA levels in shoots and leaves than in flowers, roots and fruits. *PpZML2* was preferentially expressed in young shoots but not in roots or fruits. However, some members showed similar expression levels in nearly all investigated tissues. For example, *PpJAZ1* and *PpJAZ2* showed constitutive expression features, and their expression was always maintained at high levels. In contrast, *PpJAZ6* and *PpPPD1* were always expressed at extremely low levels in all or nearly all tissues investigated.

When we examined the expression levels of these genes in different developmental stages. On the whole, the expression levels of *TIFY* members could be classified into high (TPM > 100), medium (20 < TPM < 100) and low (TPM < 20) levels. These highly expressed genes either showed constitutively high expression levels at all fruit developmental stages (*PpJAZ1* and *PpJAZ2*) or were highly expressed at both the exponential growth (stages S1 and S3) and ripening (stages S4 and S5) stages (*PpJAZ5* and *PpJAZ10*), except at the pit-hardening stage (S2). For those members with medium expression levels, including *PpJAZ3*, *PpJAZ4*, *PpJAZ7* and *PpZML1*, only *PpJAZ3* was highly expressed at the fruit ripening stages in cv. Hujingmilu (Figure 4). However, the expressional of *PpJAZ3* varied at the fruit ripening stage in different peach accessions. For example, *PpJAZ3* showed much lower expression level at the fruit ripening stage in cv. “Zao Huang Pan Tao” (Supplementary Table 6). For *TIFY* members with low expression levels, *PpJAZ6* was undetectable at the mRNA level at all fruit developmental stages, and *PpPPD1*, *PpZML2* and *PpZML4* showed extremely low expression levels throughout the entire fruit developmental stages. Interestingly, the expression of *PpJAZ8* was nearly undetectable from stage S1 to fruit ripening stages in “Hujingmilu” (Figure 4) but was active at the E1 and early S1 stages in another cultivar “Zao Huang Pan Tao” (Supplementary Table 6), indicating the relevance of this gene to the fruit set process. These results indicated that the mRNA levels of *JAZ*, *PPD* and *ZML* genes did not seem to be affected by fruit ripening cues.

**FIGURE 5:**
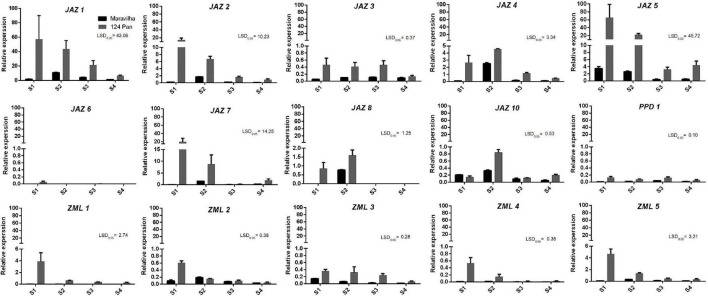
Expression profiles of the peach *JAZ*, *PPD* or *ZML* genes in fruits of “Maravilha” and “124 Pan” throughout fruit development. The *x*-axis “S1,” “S2,” “S3,” and “S4” represent different fruit developmental stages. S1, the first exponential growth stage; S2, the pit hardening stage; S3, the second exponential growth; S4, fruit ripening stage. The sampling times for stage S1 of “Maravilha” and “124 Pan” (15 days after full blooming) were the same with that of “Zao Huang Pan Tao” (Guo et al., 2018), but much earlier than that of “Hujingmilu” (34 days after full blooming, Cao et al., 2019). The *y*-axis represents the relative expression of *TIFY* members to the internal reference gene *PpTEF2*. The error bars represent ± SE of three biological replicates. Any difference between two group means greater than LSD_0.05_ is considered significantly different.

To further confirm the expression patterns of the genes during fruit developmental and fruit ripening stages, RT–qPCR was conducted with two different peach cultivars, “Maravilha” and “124 Pan” (Figure 5). Overall, the RT–qPCR results were consistent with the RNA-Seq results mentioned above. The most abundantly expressed genes, *PpJAZ1*, *PpJAZ2* and *PpJAZ5*, showed the highest expression levels in the RT–qPCR tests. Similar to the RNA-Seq results, the expression of *PpJAZ6* was nearly undetectable in fruits of both cultivars (Figure 5). A slight difference from the RNA-Seq results was that the expression of *PpJAZ8* was also detected at stage S2 (Figure 5), probably due to the variation in fruit collection time and fruit developmental process in different accessions.

In addition, all ZMLs showed relatively medium or low expression levels, which also agreed with the heatmap results. Many of the genes showed relatively higher expression levels in juvenile fruits (stages S1 and S2) than in the pre-ripening and ripening fruits. For instance, the expression levels of all *ZML* genes showed a decreasing trend during fruit development and ripening processes. Nevertheless, the gene expression patterns of these genes varied between the two cultivars. For most genes investigated, their expression levels were higher in “124 Pan” than in “Maravilha,” indicating the divergence of *JAZ*s in the fruit developmental process on different genetic backgrounds.

### Expression Profiling of Peach *JAZ*, *PPD*, and *ZML* Genes Under Exogenous MeJA Treatment

**FIGURE 6:**
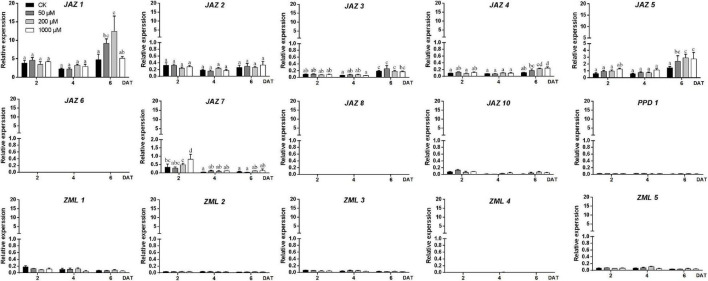
Expression profiles of the peach *JAZ*, *PPD* or *ZML* genes in the epicarp of cv. “Zhong You 18” under exogenous MeJA treatment. The *x*-axis represents days after treatment (DAT). The *y*-axis represents the relative expression of *TIFY* members to the internal reference gene *PpTEF2*. The error bars represent ± SE of three biological replicates. Significant difference at the 0.05 level is indicted by different lowercase letters based on a one-way ANOVA/Duncan’s test.

To test the effect of MeJA on different peach cultivars, three cultivars, “Huang Jin Mi,” “Zhong You 18,” and “Qing Feng,” showing no red pigmentation in fruit skin at stage S3 were selected for the exogenous MeJA (50, 200, and 1,000 μM) treatment experiments. The skin color of “Zhong You 18,” was notably affected by exogenous MeJA treatment (Supplementary Figures 2A,B), along with the significantly increased anthocyanin content (Supplementary Figure 2C). Therefore, fruits of “Zhong You 18” were collected to study the effects of MeJA on the expression levels of peach *JAZ*, *PPD*, and *ZML* genes 2, 4, and 6 days after treatment (DAT). As shown in Figure 6 and Supplementary Figure 2D, *PpJAZ6*, *PpJAZ8*, *PpPPD1*, *PpZML2*, and *PpZML4* showed no or nearly no expression in this experiment. *PpJAZ10*, *PpZML1*, *PpZML3*, and *PpZML5*, with average relative expression values below 0.2, were characterized as genes with low expression. Only the genes with the average relative expression values higher than 0.2 (including *PpJAZ1, PpJAZ2*, *PpJAZ3*, *PpJAZ4*, *PpJAZ5*, and *PpJAZ7*) were submitted for one-way ANOVA (Figure 6). Overall, most of the highly expressed genes showed higher expression levels at 6 DAT than at 2 or 4 DAT (Figure 6). However, *PpJAZ2* showed no significant changes in mRNA abundance after exogenous MeJA treatment, and the expression level of *PpJAZ7* was significantly lower at 4 or 6 DAT than at 2 DAT (Figure 6). When comparing the effects of different concentrations of exogenous MeJA with the control check (CK), significant differences were found at 2 DAT for *PpJAZ7* but not found at 4 or 6 DAT. Moreover, no significant expression level changes were found for *PpJAZ3* between the treatment groups and the CK at 6 DAT, which indicated that *PpJAZ3* might be not affected at the mRNA level under exogenous MeJA treatment. Nevertheless, the remaining three *JAZ*s, *PpJAZ1*, *PpJAZ4* and *PpJAZ5*, which showed upregulated expression levels at 6 DAT compared with at 2 or 4 DAT, were also significantly upregulated at the mRNA level under exogenous MeJA treatment compared with CK at 6 DAT, although they were differently affected by different MeJA concentrations (Figure 6). For example, the mRNA abundance of *PpJAZ1* was significantly affected by exogenous treatment with 50 μM and 200 μM MeJA compared with CK at 6 DAT; however, the effective MeJA concentrations for *PpJAZ4* were 200 and 1000 μM. All the tested concentrations of MeJA (50, 200, and 1000 μM) had significant effects on the expression level of *PpJAZ5* at 6 DAT (Figure 6). Overall, four *TIFY* members, *PpJAZ1*, *PpJAZ4*, *PpJAZ5* and *PpJAZ7*, were significantly affected at the mRNA level by exogenous MeJA treatment in the peach epicarp.

Pearson correlation analysis of anthocyanin contents and gene expression levels of *TIFY* family members under exogenous MeJA treatment was conducted (Supplementary Table 7). The results showed that the expression levels of *PpJAZ1*, *PpJAZ3*, *PpJAZ4*, and *PpJAZ5* positively correlated with anthocyanin contents at a significant level. In contrast, the expression levels of *PpJAZ7*, *PpZML1* and *PpZML3* negatively correlated with anthocyanin contents with significant *p*-value. Combined with the previous one-way ANOVA results, it was hypothesized that *PpJAZ1*, *PpJAZ4*, and *PpJAZ5* were likely induced at the mRNA level by exogenous MeJA treatment, and were involved in JA signal transduction and the regulation of secondary metabolite biosynthesis, such as anthocyanins. Nevertheless, according to a previous report, *JAZ*s respond to JA stimulation mainly at the protein level (Howe and Yoshida, 2019); therefore, it would also be possible for the other TIFY members to play important roles in the JA signaling pathway in peach fruits.

### Protein Interactions of Peach JAZs or ZMLs

**FIGURE 7:**
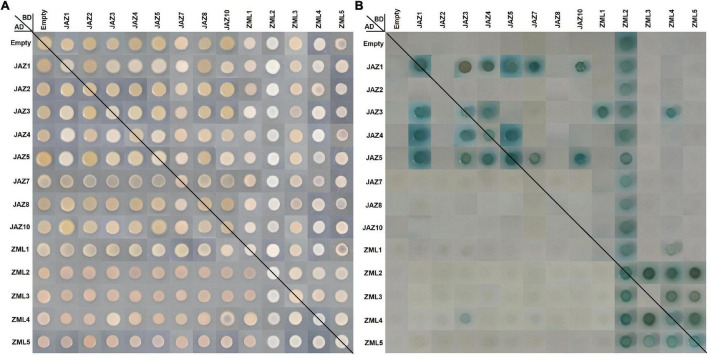
Protein-protein interactions between peach JAZ and/or ZML proteins by yeast two-hybrid assay. The positive hybrid yeast cells were grown on SD-Trp-Leu medium **(A)** and SD-Trp-Leu-His-Ade + AbA medium supplemented with X-α-Gal **(B)** to check the interactions. For more details see the Section “Materials and Methods.”

The TIFY (ZIM) domains participate in mediating the homo- and heteromeric interactions between JAZs (Chini et al., 2009), which are also included in the peach PPD and ZML subfamily members (Figure 3). To reveal the protein interaction abilities of peach JAZs and ZMLs, yeast two-hybrid analysis was conducted for the 13 members we cloned, except PpPPD1 and PpJAZ6 (Figure 7). To investigate whether these genes exhibit auto-activation activity, the full-length CDSs of the 13 genes were inserted into the yeast *pGBKT7* vector and transformed into the “Y2Hgold” strain. Yeast hybrids of these yeast transformants with the yeast strain “Y187” transformed with empty *pGADT7* vector were conducted, and the final results showed that only PpZML2 had auto-activation activity among the 13 members (Figure 7).

Yeast hybrids were conducted between these JAZs and ZMLs pair by pair. All diploid hybrids grew on DDO (SD-Trp-Leu) medium, but only those containing positive dimers grew on QDO/A/X (SD-Trp-Leu-His-Ade/AbA/X-α-Gal) medium. First, we focused on the homodimers. In total, 6 homodimers were found, including PpJAZ1, PpJAZ3, PpJAZ4, PpJAZ5, PpZML4 and PpZML5. Next, for the heterodimers, we found that JAZ proteins seldom interacted with ZMLs. JAZ-JAZ heterodimers, including PpJAZ1/3, PpJAZ1/4, PpJAZ1/5, PpJAZ1/7, PpJAZ3/4 and PpJAZ4/5, were found in reciprocal transformations (i.e., with the pair of constructs either as prey or as bait) of Y2H assays (e.g., PpJAZ1BD × PpJAZ3AD and PpJAZ3BD × PpJAZ1AD). The combinations of “PpJAZ10BD × PpJAZ1AD,” “PpJAZ7BD × PpJAZ5AD,” and “PpJAZ10BD × PpJAZ5AD” were found to be positive in one-way Y2H tests (Figure 7), which was similar to reports in other species, such as *Arabidopsis* (Chini et al., 2009), *Hevea brasiliensis* (Hong et al., 2015), cotton (Li et al., 2017), and *Mentha canadensis* (Xu et al., 2021). For the ZMLs, PpZML3/4, PpZML3/5 and PpZML4/5 were able to form heterodimers, which were confirmed by reciprocal Y2H assays, whereas “PpZML4BD × PpZML1AD” was found to be positive in the one-way Y2H test. It is worth mentioning that Y2H tests of “PpZML3BD × PpZML2AD,” “PpZML4BD × PpZML2AD,” and “PpZML5BD × PpZML2AD” were positive but could not be verified in the opposite direction due to the auto-activation activity of PpZML2. Finally, we found an interesting result in which a protein interaction between PpJAZ3 and PpZML4 was also detected in reciprocal Y2H assay. This result indicated that JAZ and ZML proteins are also able to form heterodimers.

### Protein Interactions of Peach JAZs and ZMLs With *PpMYC2*

**FIGURE 8:**
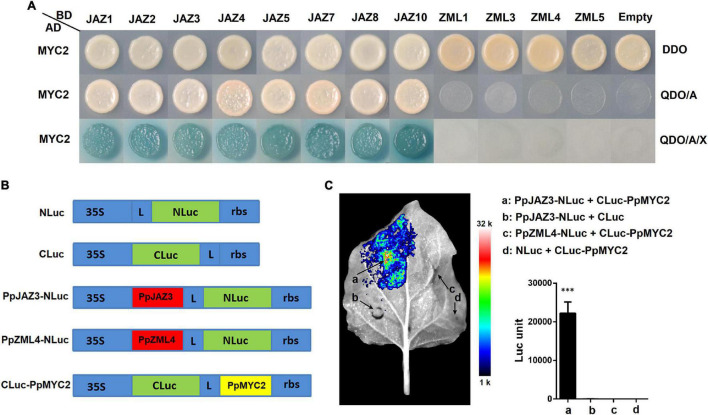
Protein-protein interactions between peach JAZ or ZML proteins with MYC2 transcription factor. **(A)** Tests of protein interactions by yeast two hybrid system. The positive hybrid yeast cells were grown on SD-Trp-Leu (DDO), SD-Trp-Leu-His-Ade + AbA (QDO/A) and SD-Trp-Leu-His-Ade + AbA media supplemented with X-α-Gal (QDO/A/X) to check the interactions. For more details see the Materials and Methods section. **(B)** Schematic diagram of the NLuc, CLuc, and NLuc/CLuc derived constructs. L, Gly/Ser linker; rbs, Transcription terminator derived from the Rubisco small subunit gene. The diagram is not drawn to scale. **(C)** Validation of the protein interaction between PpJAZ3 and PpMYC2 by split firefly luciferase complementation assay. Interactions of four pairs of the NLuc/CLuc derived constructs including “PpJAZ3-NLuc + CLuc-PpMYC2” **(a)**, “PpJAZ3-NLuc + CLuc” **(b)**, “PpZML4-NLuc + CLuc-PpMYC2” **(c)** and “NLuc + CLuc-PpMYC2” **(d)** were testified by Luc imaging and measurement of the absolute firefly luciferase (Luc) activities. The pseudocolor bar shows the range of luminescence intensity. The error bars represent ± SE of three biological replicates. ^***^P < 0.001 (Student’s *t*-test).

In Arabidopsis, AtJAZs function as repressors of JA signaling by interacting with AtMYC2 (Pauwels and Goossens, 2011). The sequence of AtMYC2 was used as a query to blast against the peach genome to search for its homologs. One peach gene, *Prupe.5G035400*, that showed the highest similarity with *AtMYC2* was cloned and designated *PpMYC2*. Deduced amino acid sequence alignment of PpMYC2, AtMYC2 and AtMYC2 homologs from apple, pear, strawberry and tomato showed that these MYCs not only shared conserved sequences at the MYC N-terminal region and bHLH domain, but also at the C-terminus (Supplementary Figure 3), implying possibly conserved protein functions. To investigate whether JAZ-MYC2 dimers also exist in peach, yeast two-hybrid-based protein interaction tests were conducted between PpJAZs and PpMYC2 and between PpZMLs and PpMYC2 (Figure 8A). JAZs (not including PpJAZ6) and ZMLs (not including PpZML2 for its auto-activity) were fused with GAL4BD as the bait, and PpMYC2 was fused with GAL4AD as the prey. The empty vectors were used as the controls. After hybridization, all positive diploid transformants for each combination of JAZ/ZML-MYC2 grew well on DDO medium but showed divergent growth status on QDO/A or QDO/A/X media, which indicated that all JAZs tested interacted with PpMYC2, but none of the ZMLs was able to bind with PpMYC2, very likely due to the lack of the Jas domain.

To further validate the protein interactions between JAZs and MYC2 of peach, PpJAZ3 and PpZML4 were selected to test the interaction with PpMYC2 using the split firefly luciferase complementation assay (Figure 8B). Transient over-expression of “PpJAZ3-NLuc + CLuc-PpMYC2” structures combination in tobacco leaves was able to rescue an intense luciferase activity, which was not exhibited in the combination “PpZML4-NLuc + CLuc-PpMYC2” and the empty control groups “PpJAZ3-NLuc + CLuc” and “NLuc + CLuc-PpMYC2” (Figure 8C). The rescued luciferase activity by co-expression of PpJAZ3-NLuc and CLuc-PpMYC2 was also validated by measurement of firefly luciferase activities using a luminometer (Figure 8C). These results indicated that PpJAZ3 but not PpZML4 could interact with PpMYC2, which was in line with the Y2H results.

## Discussion

The TIFY family is a large plant-specific gene family characterized by the TIFY domain. The TIFY domain widely exists from liverworts to dicots but not in green algae (Bai et al., 2011; Garrido-Bigotes et al., 2019). TIFY TFs are classified into four subfamilies, *TIFY*, *JAZ*, *PPD* and *ZML*, according to their different domain and motif architectures; however, not all subfamilies exist in each species. For example, the *TIFY* and *PPD* subfamilies are not found in some monocots (Bai et al., 2011). In this study, we found that like other dicot plants, peach contains members of all four subfamilies but also shows specific characteristics in terms of gene numbers and duplication patterns. Gene numbers of each subfamily varied among different species due to segmental or tandem duplication and gene loss events. Similar to the other dicot plants and mosses, peach has more JAZ members than ZMLs (Figure 2). Interestingly, peach and moss shared close evolutionary relationships for ZMLs but relatively large phylogenetic distances for JAZs (Figure 2). These features of JAZs are in line with their redundancy by gene duplication and functional divergence due to the emergence of new motifs (Chini et al., 2009; Garrido-Bigotes et al., 2019). Moreover, like *Vitis vinifera*, peach did not experience additional recent whole-genome duplication after the gamma (γ) whole-genome triplication event in the ancestor of core eudicots (Jiao et al., 2012; Verde et al., 2013); therefore, it is able to deduce the possible small segmental or tandem duplication and gene loss events by comparing the gene numbers and positions of the two species. According to previous reports, there are 2 *TIFY* and 2 *PPD* members in the grape genome (Bai et al., 2011; Zhang et al., 2012); however, only 1 TIFY and 1 PPD exist in the peach genome, demonstrating the occurrence of gene loss events for these two subfamilies after the divergence of *Vitaceae* and *Rosaceae*. Additionally, considering the recent whole-genome duplication event during the evolutionary split of the genera *Malus* and *Prunus*, it is also meaningful to compare the gene numbers between peach and apple. Interestingly, 8 TIFY subfamily members were found in the apple genome, demonstrating that the *TIFY* subfamily experienced an apparent expansion event after the recent whole-genome duplication in the apple genome compared to the peach genome (Figure 1C). There were 9, 11, and 18 JAZ subfamily members in peach, grape and apple, respectively, indicating important roles of both gene loss and whole genome duplication in the JAZ subfamily of *Rosaceae* (Figure 2).

JAZ proteins function as repressors of JA signaling and are degraded by the 26S proteasome *via* interaction with the F-box subunit of the SCF*^COI^*^1^ ubiquitin ligase (Howe and Yoshida, 2019). Therefore, JAZs respond to JA stimulation mainly at the protein level. However, some reports have shown that the response also occurs at the transcriptional level. For example, following treatment of leaves with MeJA, most JAZs transcriptionally changed in apple and tomato (Li et al., 2015; Chini et al., 2017). However, gene expression changes in *TIFY* family members in fruit under MeJA treatment have rarely been reported. One study in strawberry fruit reported that two *JAZ*s, *FaJAZ1* and *FaJAZ8.1*, were only upregulated at 30 min and 6 h, respectively, under 100 μM MeJA treatment but not at other time points (Garrido-Bigotes et al., 2018). In this study, we researched the gene expression changes of 15 *JAZ*, *PPD* and *ZML* subfamily members and found that only 4 of them were significantly influenced by exogenous treatment with MeJA (Figure 6). Among them, *PpJAZ1*, *PpJAZ4* and *PpJAZ5* were likely stimulated by MeJA and positively correlated with fruit epicarp pigmentation. In contrast, *PpZML1* and *PpZML3* showed a negative correlation with fruit epicarp anthocyanin contents and were reduced at the mRNA level under exogenous treatment (Figure 6 and Supplementary Table 7). The potential involvement of these *TIFY* genes in JA-induced anthocyanin accumulation at the transcriptional level may enrich the present model in which anthocyanin activators are hampered or degraded by protein interactions with JAZ repressors (Qi et al., 2011). Future research is needed to understand the relationship of JA signaling and anthocyanin biosynthesis in peach fruit. Moreover, similar to *FaJAZ1* and *FaJAZ8.1*, most of these affected genes showed variation in expression levels only at specific MeJA concentrations and time points. For example, Expression level of *PpJAZ4* was significantly affected at 6 days under 200 or 1000 μM MeJA but not at the other concentrations and time points (Figure 6). Considering the critical roles of leaves in defense and stress responses, we hypothesize that the gene expression of *TIFY* family members in fruit tissues is less sensitive to MeJA than that of leaves, which needs to be addressed by further studies.

JAZ proteins usually form homo- or heterodimers *via* the TIFY domain. The genome-wide interactions of JAZs have been verified in *Arabidopsis*, *Hevea brasiliensis*, cotton and *Mentha canadensis* by yeast two-hybrid assays in previous reports (Chini et al., 2009; Hong et al., 2015; Li et al., 2017; Xu et al., 2021). Some JAZs (such as AtJAZ1, AtJAZ3, AtJAZ4 and AtJAZ9 of *Arabidopsis*, GhJAZ1-D, GhJAZ2-A and GhJAZ13-D of cotton, McJAZ2, McJAZ3 and McJAZ5 of *Mentha canadensis*) participate in both homodimers and heterodimer formation. One hypothesis suggests that dimerization may contribute to strengthening the functions of JAZs by facilitating the stability of JAZ proteins (He et al., 2020). Nevertheless, some members cannot form dimers with themselves or other JAZs, such as AtJAZ2, AtJAZ5, AtJAZ7, AtJAZ10, AtJAZ11 and AtJAZ12 (Chini et al., 2009), which also play important roles in JA signaling. Therefore, the dimers of JAZs may play other important roles in other aspects in addition to protein stability. Another hypothesis suggests that the homodimers of JAZs may contribute to the stability of proteins, and the heterodimers also facilitate the simultaneous interaction of multiple JAZs with MYC proteins (Chini et al., 2009; Geerinck et al., 2010), which increases the complexity of the JAZ-MYC complex to deal with different situations. Indeed, in this study, we also identified peach JAZ dimers by Y2H and found similar interaction features with Arabidopsis and cotton, such as JAZ members (PpJAZ1, PpJAZ3, PpJAZ4 and PpJAZ5) participating in both homo- and heterodimer formation and members (PpJAZ2, PpJAZ8 and PpJAZ10) not involved in dimers (Figure 7). These findings established JAZ interaction networks in peach, and heterodimer information would be useful for understanding the complexity of the JAZ-MYC complex. Furthermore, considering the relative conservation of the TIFY domain between the JAZ and ZML subfamilies, we extended the JAZ-JAZ interaction to the ZML-ZML and JAZ-ZML interactions and discovered 2 homodimers and 7 heterodimers for ZMLs and the only interesting JAZ-ZML dimer, PpJAZ3-PpZML4. Unlike JAZs, ZMLs do not have the Jas domain which is necessary for the interaction with COI1 and therefore, cannot be degraded by the 26S proteasome. In a previous report, AtSPL9 could interact with the TIFY domain to inhibit the degradation of JAZs by interfering with the JAZ-COl1 interaction (Mao et al., 2017). Therefore, the JAZ-ZML interaction may inhibit degradation of JAZ by interfering with COI1 activity. To date, the PpJAZ3-PpZML4 dimer is the first JAZ-ZML dimer found in plants, suggesting the possible role of the ZML-JAZ-MYC complex in JA signaling.

## Conclusion

In this study, 16 *TIFY* family genes from the peach genome were identified according to the peach reference genome sequence information and further validated by cloning and sequencing. Syntenic analysis showed that both tandem and segmental gene duplication played important roles in shaping the expansion pattern of this gene family. The phylogenetics, location, structure, and conserved domains and motifs of these genes were analyzed, and finally, the peach TIFY family was characterized into 9 *JAZ*, 1 *TIFY*, 1 *PPD* and 5 *ZML* subfamily members. Expression profile analyses showed that each *JAZ*, *PPD*, and *ZML* gene exerted limited effects on fruit ripening cues; however, 4 of the genes in fruit skin were affected by exogenous treatment with MeJA at the transcriptional level. In addition, the *TIFY* family member protein interaction networks established by yeast two-hybrid (Y2H) assays showed 6 pairs of homodimers and 16 pairs of heterodimers. The PpJAZ3-PpZML4 interaction found in this study suggests the potential formation of the ZML-JAZ-MYC complex in the JA signaling pathway, which may extend our knowledge of this gene family’s function in diverse biological processes.

## Data Availability Statement

The datasets presented in this study can be found in online repositories. The names of the repository/repositories and accession number(s) can be found in the article/Supplementary Material.

## Author Contributions

HZ, YS, and JZ conceived and designed the experiments. YS, HZ, HY, HP, KQ, QX, and HC performed the experiments. HZ and YS wrote the manuscript. HP, SF, and JZ revised the manuscript. All authors contributed to the article and approved the submitted version.

## Conflict of Interest

The authors declare that the research was conducted in the absence of any commercial or financial relationships that could be construed as a potential conflict of interest.

## Publisher’s Note

All claims expressed in this article are solely those of the authors and do not necessarily represent those of their affiliated organizations, or those of the publisher, the editors and the reviewers. Any product that may be evaluated in this article, or claim that may be made by its manufacturer, is not guaranteed or endorsed by the publisher.

## Footnotes

1
http://smart.embl.de/

2
https://www.ncbi.nlm.nih.gov/cdd/

3
https://www.rosaceae.org/species/prunus_persica/genome_v2.0.a1

4
https://phytozome-next.jgi.doe.gov/

5
https://itol.embl.de

6
https://www.ncbi.nlm.nih.gov/sra

## Supplementary Material

Supplementary Figure 1 Partial amino acid sequence alignments of the JAZ, PPD and ZML subfamily members of peach and Arabidopsis.

Supplementary Figure 2 Exogenous MeJA treatment of fruits of cv. Zhong You 18. **(A)** Fruit epicarp of “Zhong You 18” at stage S3 showed no red pigmentation before MeJA treatment. **(B)** Coloration of fruit skins was affected by exogenous MeJA treatment. **(C)** Anthocyanin content of the fruit skins after MeJA treatment. The error bars represent ± SE of three biological replicates. **P* < 0.05 (Student’s *t*-test). **(D)** The background gene expression levels of TIFY family genes in the epicarp of “Zhong You 18” fruits at stage S3 before MeJA treatment. For more details see the Section “Materials and Methods”.

Supplementary Figure 3 Alignment of the deduced MYC2 amino acid sequences. The following GenBank or genome sequencing project accession numbers were used: *Arabidopsis thaliana* AtMYC2 (At1g32640); *Malus* × *domestica* MdMYC2 (MDP0000136498); *Fragaria vesca* FvMYC2 (FvH4_7g17380.1); *Solanum lycopersicum* SlMYC2 (Solyc08g076930); and *Pyrus pyrifolia* PpyMYC2 (Ppy06g0316.1).

Supplementary Table 1 Primers used in this study.

Supplementary Table 2 Hits obtained by searching the peach genome v2.0 sequences using the Hmmsearch software of the HMMER suite. Hidden Markov model (HMM) profiles of the TIFY domain, the Jas (CCT-2) motif and the CCT motif were extracted from the Pfam database (accession numbers PF06200, PF09425, and PF06203, respectively).

Supplementary Table 3 Scanning of the conserved domains of the JAZ, PPD and ZML subfamily members in the SMART database.

Supplementary Table 4 Comparative syntenic analysis between peach and apple genomes.

Supplementary Table 5 Expression levels (TPM) of TIFY family genes that derived from RNA-Seq data downloaded from NCBI SRA database. Average and standard derivation (SD) of the means were calculated using three biological replicates. For more details see the section “Materials and Methods”.

Supplementary Table 6 Expression levels (TPM) of TIFY family genes that derived from RNA-Seq data downloaded from NCBI SRA database [PRJNA401307, cv. Zao Huang Pan Tao (ZH); E, 0 DAFB; S1, 15 DAFB; S4, 65 DAFB].

Supplementary Table 7 Pearson correlation analysis of anthocyanin contents and gene expression levels of TIFY family members under exogenous MeJA treatment. For the genes with extremely low expression levels, this analysis was not applied (NA).

Supplementary Data Sheet 1 Deduced protein sequences alignment of *TIFY* family genes from various plant species.
