# Supplementary figures and images for: Genome-Wide Analysis of the Gene Structure, Expression and Protein Interactions of the Peach (Prunus persica) TIFY Gene Family
Source: Front Plant Sci. 2022 Feb 17;13:792802. doi: 10.3389/fpls.2022.792802 (PMC8891376; doi:10.3389/fpls.2022.792802)

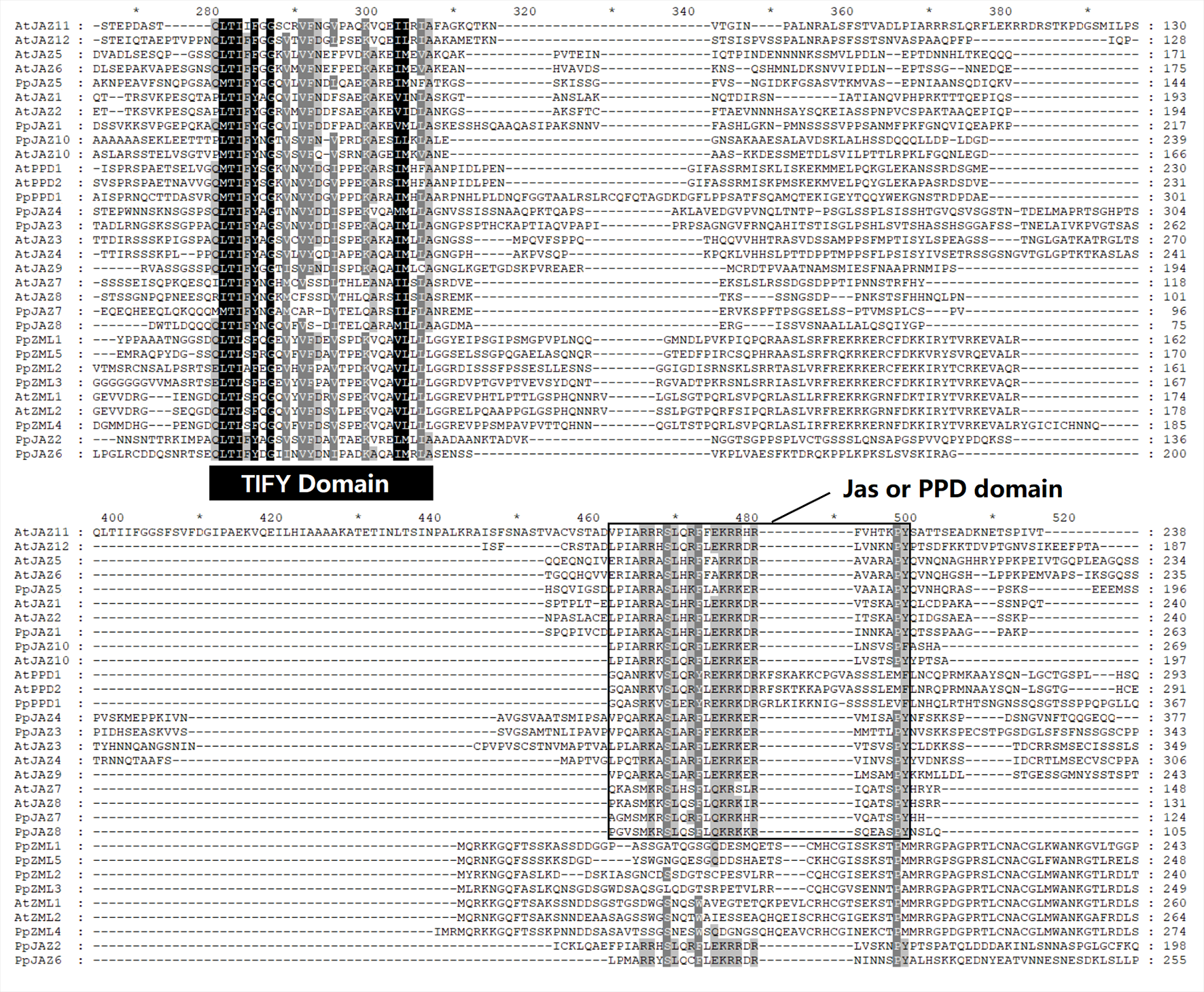

Supplement: Supplementary Figure 1 — Partial amino acid sequence alignments of the JAZ, PPD and ZML subfamily members of peach and Arabidopsis. [file Image_1.tif]

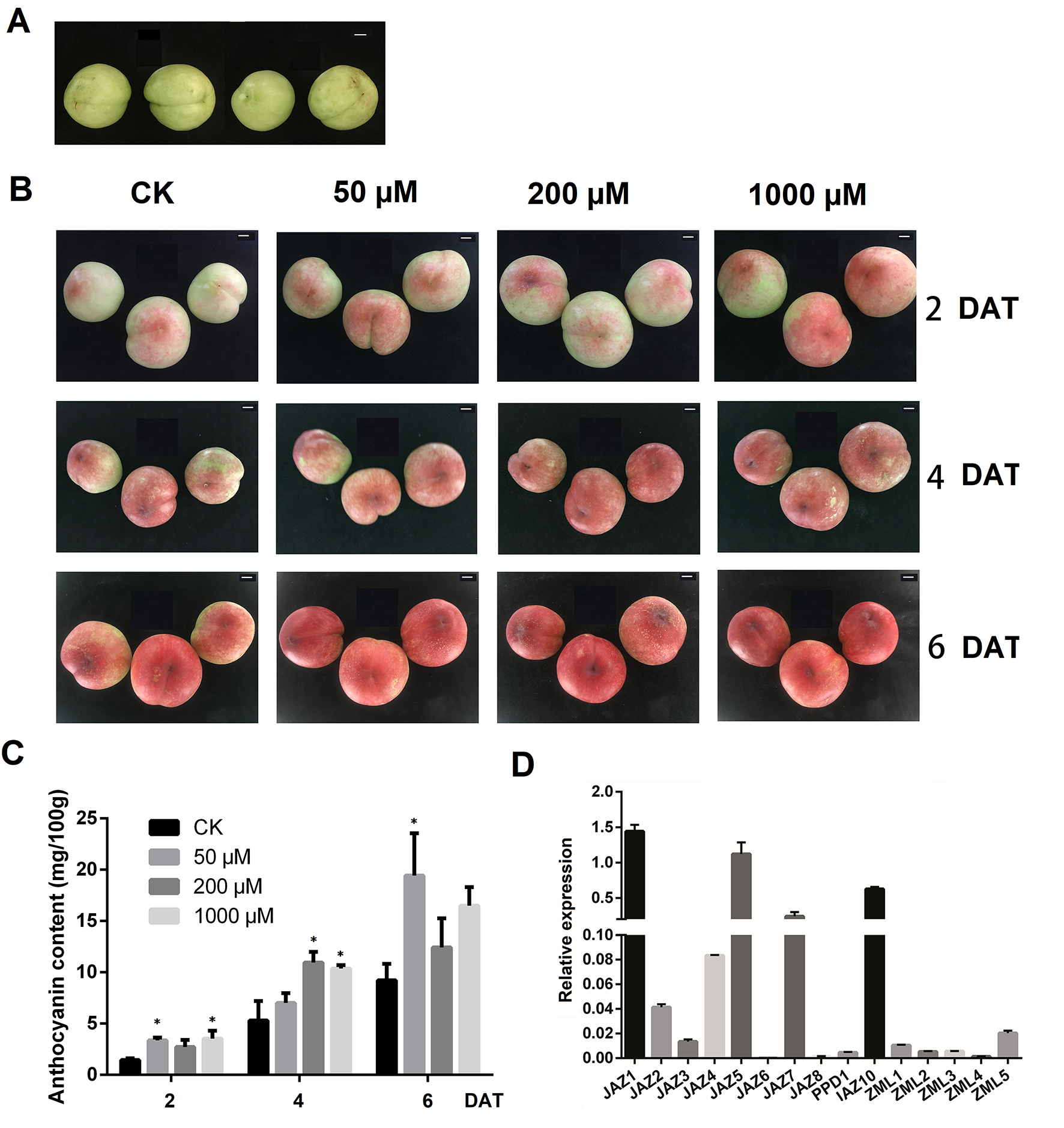

Supplement: Supplementary Figure 2 — Exogenous MeJA treatment of fruits of cv. Zhong You 18. (A) Fruit epicarp of “Zhong You 18” at stage S3 showed no red pigmentation before MeJA treatment. (B) Coloration of fruit skins was affected by exogenous MeJA treatment. (C) Anthocyanin content of the fruit skins after MeJA treatment. The error bars represent ± SE of three biological replicates. *P < 0.05 (Student’s t-test). (D) The background gene expression levels of TIFY family genes in the epicarp of “Zhong You 18” fruits at stage S3 before MeJA treatment. For more details see the Section “Materials and Methods”. [file Image_2.tif]

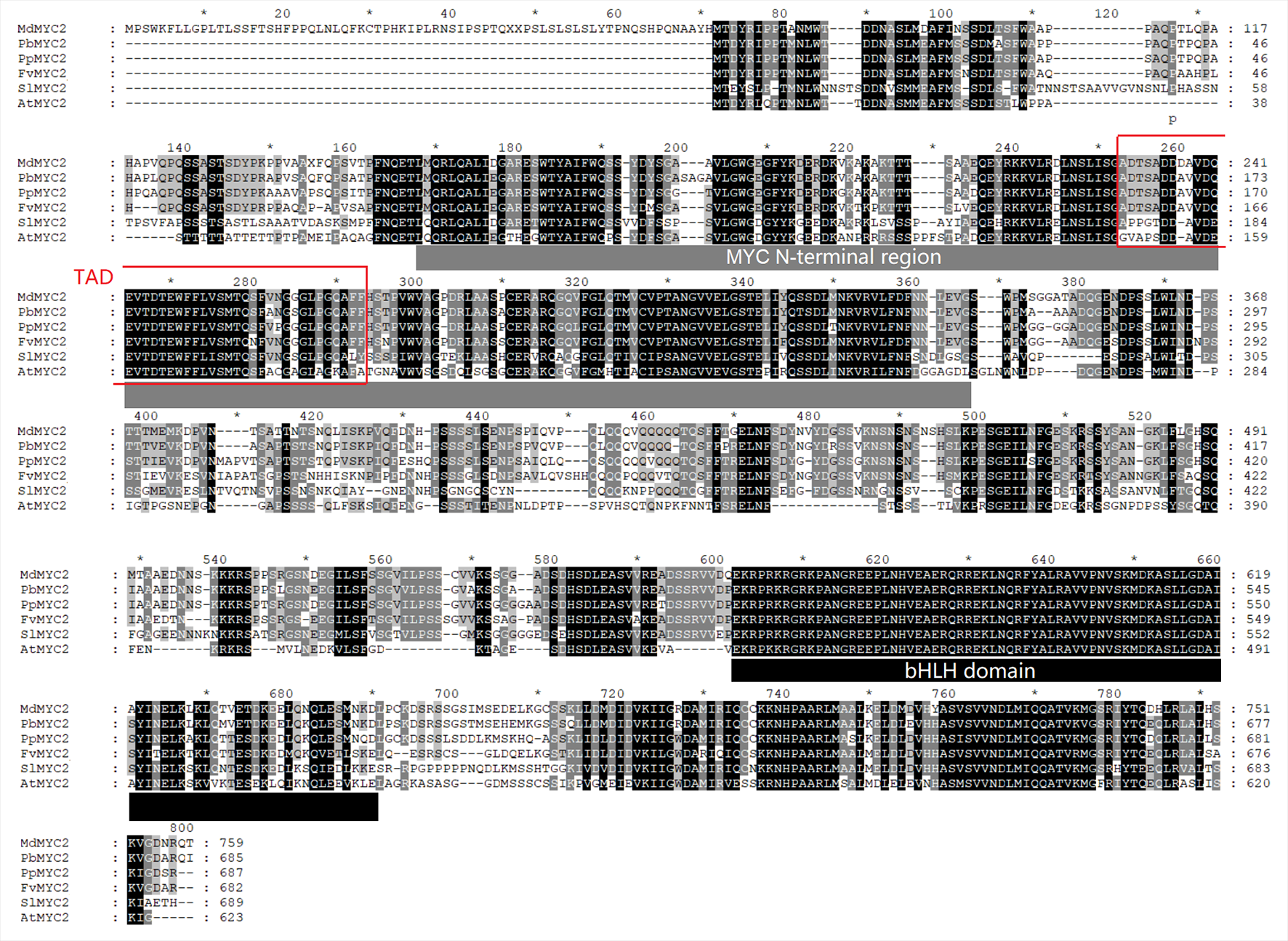

Supplement: Supplementary Figure 3 — Alignment of the deduced MYC2 amino acid sequences. The following GenBank or genome sequencing project accession numbers were used: Arabidopsis thaliana AtMYC2 (At1g32640); Malus × domestica MdMYC2 (MDP0000136498); Fragaria vesca FvMYC2 (FvH4_7g17380.1); Solanum lycopersicum SlMYC2 (Solyc08g076930); and Pyrus pyrifolia PpyMYC2 (Ppy06g0316.1). [file Image_3.tif]
