# Supplementary material for: Genome-Wide Analysis of the Gene Structure, Expression and Protein Interactions of the Peach (Prunus persica) TIFY Gene Family
Source: Front Plant Sci. 2022 Feb 17;13:792802. doi: 10.3389/fpls.2022.792802 (PMC8891376; doi:10.3389/fpls.2022.792802)
Supplement: Supplementary Table 1 — Primers used in this study. [file Table_1.DOC]

Table S1. Sequences of primers used for cloning genes and vectors construction and qRT-PCR

| Primer name | Sequence | Description |
| --- | --- | --- |
| JAZ 1-Q-F | AACCCATTGTTTGTGATCTACCG | Quantitative RT-PCR analysis |
| JAZ 1-Q-R | TGGTGCTTTGTTGTTGATCCTATC | Quantitative RT-PCR analysis |
| JAZ 2-Q-F | AACAGCAACACCACCAGAAAGA | Quantitative RT-PCR analysis |
| JAZ 2-Q-R | AACTGCATCAAAGACGCTAACAC | Quantitative RT-PCR analysis |
| JAZ 3-Q-F | CCGCTGATCTCAGGAATGGA | Quantitative RT-PCR analysis |
| JAZ 3-Q-R | CCAGCCAGCAACATAATAGCCT | Quantitative RT-PCR analysis |
| JAZ 4-Q-F | TGCAAAGACTCAGGGTATGCTAG | Quantitative RT-PCR analysis |
| JAZ 4-Q-R | TGAAAGGCATCAAATGAGGAAGT | Quantitative RT-PCR analysis |
| JAZ 5-Q-F | ACAAATTCGGTTCTGCCTCTG | Quantitative RT-PCR analysis |
| JAZ 5-Q-R | TGCCACCCTTTCTTTCCTCT | Quantitative RT-PCR analysis |
| JAZ 6-Q-F | GCTTCCTGGATTGCGATGTG | Quantitative RT-PCR analysis |
| JAZ 6-Q-R | ACAAGAGGTTTGACCGATGAGTT | Quantitative RT-PCR analysis |
| JAZ 7-Q-F | AGCGATGTGCGCTCGTGA | Quantitative RT-PCR analysis |
| JAZ 7-Q-R | TGCCAGCAACAGGGCTACAC | Quantitative RT-PCR analysis |
| JAZ 8-Q-F | TGGGCGACTGGACTCTGG | Quantitative RT-PCR analysis |
| JAZ 8-Q-R | AAGCAACGCTGCGTTTGAC | Quantitative RT-PCR analysis |
| JAZ 10-Q-F | AAGTCGCTCCTCCAACCTCC | Quantitative RT-PCR analysis |
| JAZ 10-Q-R | GCTGATCCATGCCGAAGAAAT | Quantitative RT-PCR analysis |
| PPD 1-Q-F | GGATTGTCGTTTCGCCTCA | Quantitative RT-PCR analysis |
| PPD 1-Q-R | CACGGAAACCTGGACATCG | Quantitative RT-PCR analysis |
| ZML 1-Q-F | GGGAGTTCTAACAGGTGGTCCTA | Quantitative RT-PCR analysis |
| ZML 1-Q-R | AACTGAGTCTTTAGCTTCGCCAT | Quantitative RT-PCR analysis |
| ZML 2-Q-F | GGGTATAGGCGACATCTCACG | Quantitative RT-PCR analysis |
| ZML 2-Q-R | TCGTCCTTCAATGATGCAAACT | Quantitative RT-PCR analysis |
| ZML 3-Q-F | CCTGAGAAGGTGCAAGCAGTAC | Quantitative RT-PCR analysis |
| ZML 3-Q-R | CACGAAACCGAACCAGTGAAG | Quantitative RT-PCR analysis |
| ZML 4-Q-F | CTTATGTGGGCAAACAAGGGTA | Quantitative RT-PCR analysis |
| ZML 4-Q-R | CGTTCTTGCTTACGGAAGGGTT | Quantitative RT-PCR analysis |
| ZML 5-Q-F | CCGCCAAGAAGTTGCACTCA | Quantitative RT-PCR analysis |
| ZML 5-Q-R | TGGCTATCATCCTGCCCTGA | Quantitative RT-PCR analysis |
| JAZ 1-BD/AD-F | ggaattccatatgATGTCGAGTTCATCGGAGACTCTG | *Nde I* for pGBKT7/pGADT7 |
| JAZ 1-BD-R | acgcgtcgacTCATTGGGTTGGTTGAGCAGC | *Sal I* for pGBKT7 |
| JAZ 1-AD-R | ccgctcgagTCATTGGGTTGGTTGAGCAGC | *Xho I* for pGADT7 |
| JAZ 2-BD/AD-F | ggaattccatatgATGGAAGAAAAATCTCAAGCTGG | *Nde I* for pGBKT7/pGADT7 |
| JAZ 2-BD-R | acgcgtcgacTCAAGCTGGGTAGGCAGTAGC | *Sal I* for pGBKT7 |
| JAZ 2-AD-R | ccgctcgagTCAAGCTGGGTAGGCAGTAGC | *Xho I* for pGADT7 |
| JAZ 3-BD/AD-F | ggaattccatatgATGGAGAGGGATTTTTTGGGTT | *Nde I* for pGBKT7/pGADT7 |
| JAZ 3-BD-R | acgcgtcgacCTAATTTATAGCTTGAGGAGGGCA | *Sal I* for pGBKT7 |
| JAZ 3-AD-R | ccgctcgagCTAATTTATAGCTTGAGGAGGGCA | *Xho I* for pGADT7 |
| JAZ 4-BD/AD-F | ggaattccatatgATGGAGAGGGATTTCTTGGGTT | *Nde I* for pGBKT7/pGADT7 |
| JAZ 4-BD-R | acgcgtcgacTCATTGTTGTTCTCCCTGCTGG | *Sal I* for pGBKT7 |
| JAZ 4-AD-R | ccgctcgagTCATTGTTGTTCTCCCTGCTGG | *Xho I* for pGADT7 |
| JAZ 5-BD/AD-F | ggaattccatatgATGGCAGAGAAGGTCAATTTTGC | *Nde I* for pGBKT7/pGADT7 |
| JAZ 5-BD-R | acgcgtcgacTCAATATAGCCTGAGTTCAAGCTGC | *Sal I* for pGBKT7 |
| JAZ 5-AD-R | ccgctcgagTCAATATAGCCTGAGTTCAAGCTGC | *Xho I* for pGADT7 |
| JAZ 7-BD/AD-F | ggaattccatatgATGAGGAGAAACTGCAACCTGG | *Nde I* for pGBKT7/pGADT7 |
| JAZ 7-BD/AD-R | cgcggatccCTAGTGATGGTATGGAGATGTTGCT | *BamH I* for pGBKT7/pGADT7 |
| JAZ 8-BD/AD-F | ggaattccatatgATGCCCACTCCTTCACCGC | *Nde I* for pGBKT7/pGADT7 |
| JAZ 8-BD/AD-R | cgcggatccCTATTGTAGGCTGTTGTATGGAGAAGC | *BamH I* for pGBKT7/pGADT7 |
| JAZ 10-BD/AD-F | ggaattccatatgATGAATCTCGTGCCTCTTTTAAGA | *Nde I* for pGBKT7/pGADT7 |
| JAZ 10-BD-R | acgcgtcgacTTAGGCATGGCTGGCAAATG | *Sal I* for pGBKT7 |
| JAZ 10-AD-R | ccgctcgagTTAGGCATGGCTGGCAAATG | *Xho I* for pGADT7 |
| ZML 1-BD/AD-F | ggaattccatatgATGTCAGAATCCAATCACCAGAAC | *Nde I* for pGBKT7/pGADT7 |
| ZML 1-BD/AD-R | ccggaattcTCATAAATTCCTGTCCACAGTCATG | *EcoR I* for pGBKT7/pGADT7 |
| ZML 2-BD/AD-F | ggaattccatatgATGGAGATGGTGAACGCTCAG | *Nde*  for pGBKT7/pGADT7 |
| ZML 2-BD/AD-R | cgcggatccTCAAGTTCCTGGCCAGTGAGTC | *BamH I* for pGBKT7/pGADT7 |
| ZML 3-BD/AD-F | ggaattccatatgATGGCGGCCGTGAATCCG | *Nde I* for pGBKT7/pGADT7 |
| ZML 3-BD/AD-R | cgcggatccCTAATCAAAATTAGTAGGGATTTCTAAATCTG | *BamH I* for pGBKT7/pGADT7 |
| ZML 4-BD/AD-F | ccggaattcATGGATGACATCTGTGGCAGC | *EcoR I* for pGBKT7/pGADT7 |
| ZML 4-BD/AD-R | cgcggatccTTATAAATCAGGTTTGATATCTTCGTTC | *BamH I* for pGBKT7/pGADT7 |
| ZML 5-BD/AD-F | ggaattccatatgATGTACGGACACTCCGAGCC | *Nde I* for pGBKT7/pGADT7 |
| ZML 5-BD/AD-R | cgcggatccCTACTGTTCAGCAACTAAAGCTGAGC | *BamH I* for pGBKT7/pGADT7 |
| MYC2-AD-F | ggaattccatatgATGACGGACTATCGGATACCG | *Nde I* for pGADT7 |
| MYC2-AD-R | cgcggatccTTACCGGGAGTCGCCGAT | *BamH I* for pGADT7 |
| MYC2-CLuc-F | cgcggatccaACGGACTATCGGATACCGCCGA | *BamH I* for pCambia 1300 Cluc |
| MYC2-CLuc-R | acgcgtcgacTTACCGGGAGTCGCCGAT | *Sal I* for pCambia 1300 Cluc |
| JAZ3-NLuc-F | cgagctcATGGAGAGGGATTTTTTGGGTT | *Sac I* for pCambia 1300 Nluc |
| JAZ3-NLuc-R | acgcgtcgacATTTATAGCTTGAGGAGGGCA | *Sal I* for pCambia 1300 Nluc |
| ZML4-NLuc-F | cgcggatccATGGATGACATCTGTGGCAGC | *BamH I* for pCambia 1300 Nluc |
| ZML4-NLuc-R | acgcgtcgacTTGGTTATTATGACAAATGCAGATG | *Sal I* for pCambia 1300 Nluc |
