# Supplementary material for: Genome-Wide Analysis of the Gene Structure, Expression and Protein Interactions of the Peach (Prunus persica) TIFY Gene Family
Source: Front Plant Sci. 2022 Feb 17;13:792802. doi: 10.3389/fpls.2022.792802 (PMC8891376; doi:10.3389/fpls.2022.792802)
Supplement: Supplementary Data Sheet 1 — Deduced protein sequences alignment of TIFY family genes from various plant species. [file Data_Sheet_1.DOC]

>AtJAZ7

---------------------------------------------------------------MIIIIKNCDKPLLNFKE-------MEMQTKCDLELR--------------------------------------------------------------------------------------------------------------------------------LLTSSYDSDFHSSLDESSSSEI--------------------------------------------------------------------SQPKQESQILTIFYNGHMCVSSDLTHLE------------------------ANAILS-----L---ASR-DVE-------------------------EKSLSLRS-------------------------------SDGSDPP----TIPNNST---------------------RFHYQ--------------------------------------KASMKRSLHSFLQKRSL-RIQAT-SPYHRYR-------------------------------------------------------------------------------------------------------------------------------------------------------------------------------------------------------------------------------------------------------------------------------------------------------------------------------------------------------------------------------------------------------------------------------------

>AtJAZ8

---------------------------------------------------------------------------------------MKLQQNCDLELR--------------------------------------------------------------------------------------------------------------------------------LFPTSYDSDSSDTTSVVESTSS------------------------------------------------------------------GNPQPNEESQRITIFYNGKMCFSSDVTHLQ------------------------ARSIIS-----I---ASR-EMK-------------------------TKSSSNGS---------------------------------DPPNK----STSFHHN---------------------QLPNP--------------------------------------KASMKKSLQSFLQKRKI-RIQAT-SPYHSRR-------------------------------------------------------------------------------------------------------------------------------------------------------------------------------------------------------------------------------------------------------------------------------------------------------------------------------------------------------------------------------------------------------------------------------------

>MdJAZ3

-----------------------------------------------------------------------------------------MRRNCNLELQ-----------------------------------------------------------------------------------------------------------------LCFPGANET-------------------------------------------------------------------------------------------------QQQEQQQQMTIFYNGIACVRD-VTELQ------------------------ARSIIF-----L---ANK-EME-------------------------ERVKSPLT----------------------------------PSGS----SEPPTPTVMS------------------PLCSP------------------------------------VAGMSMKRSLQRFLQKRKH-RTQAT-SPYHH---------------------------------------------------------------------------------------------------------------------------------------------------------------------------------------------------------------------------------------------------------------------------------------------------------------------------------------------------------------------------------------------------------------------------------------

>MdJAZ4

-----------------------------------------------------------------------------------------MRRNCNLELQ-------------------------------------------------------------------------------------------------------------------------------------------LCFPGANETQQ-----------------------------------------------------------------------QEQQQQMTIFYNGIACVRD-VTELQ------------------------ARSIIF-----L---ANK-EME-------------------------ERVKSPLT----------------------------------PSGS----SEPPTPTVMS------------------PLCSP------------------------------------VAGMSMKRSLQRFLQKRKH-RTQAT-SPYHH---------------------------------------------------------------------------------------------------------------------------------------------------------------------------------------------------------------------------------------------------------------------------------------------------------------------------------------------------------------------------------------------------------------------------------------

>MdJAZ5

-----------------------------------------------------------------------------------------MRRNCNLELQ----------------------------------------------------------------------------------------------------------------------LQLH------NPSADSSFPNPNETQQQEQHEQ----------------------------------------------------------------------QQQQQQQMTIFYNRMMCVRD-VTELQ------------------------ARSIIF-----L---ANK-EME-------------------------ERVKSPFT----------------------------------PSGS----SEPXSPNLMS------------------PLGSP------------------------------------VAGTSLKRSLQRFLQERKH-RNQAT-SPYHR---------------------------------------------------------------------------------------------------------------------------------------------------------------------------------------------------------------------------------------------------------------------------------------------------------------------------------------------------------------------------------------------------------------------------------------

>MdJAZ6

-----------------------------------------------------------------------------------------MRRNCNLELQ--LQ--------------------------------------------------------------------------------------------------------------------LH------NPSADSSFPNPNETQQQEQHEQ----------------------------------------------------------------------QQQQQQQMTIFYNRMMCVRD-VTELQ------------------------ARSIIF-----L---ANK-EME-------------------------ERVKSPFT----------------------------------PSGS----SEPXSPNLMS------------------PLGSP------------------------------------VAGTSLKRSLQRFLQERKH-RNQAT-SPYHR---------------------------------------------------------------------------------------------------------------------------------------------------------------------------------------------------------------------------------------------------------------------------------------------------------------------------------------------------------------------------------------------------------------------------------------

>MdJAZ8

-----------------------------------------------------------------------------------------MRRNCNLELQ--LY-----------------------------------------------------------------------------------------------------------------------------------SPGSNETQQQEHQQQ--------------------------------------------------------------------------QQQLTIFYNGMMCVRD-VTELQ------------------------ARSILF-----L---ANK-GME-------------------------ERVKSPFT--------------------------PPSGLSEPPSPN----VVSPLCS-------------------------------------------------------------TVAGVSMKRSLQRFLQKRKH-RTQAT-SPYHR---------------------------------------------------------------------------------------------------------------------------------------------------------------------------------------------------------------------------------------------------------------------------------------------------------------------------------------------------------------------------------------------------------------------------------------

>PpJAZ7

-----------------------------------------------------------------------------------------MRRNCNLELQ--------------------------------------------------------------------------------------------------------------------------------LLHPSYETHKKQQEQEQHEEQL-----------------------------------------------------------------------QKQQQMMTIFYNGAMCARD-VTELQ------------------------ARSILF-----L---ANR-EME-------------------------ERVKSPFT--------------------------------PSGSEL----SSPTVMS---------------------PLCSP------------------------------------VAGMSMKRSLQRFLQKRKH-RVQAT-SPYHH---------------------------------------------------------------------------------------------------------------------------------------------------------------------------------------------------------------------------------------------------------------------------------------------------------------------------------------------------------------------------------------------------------------------------------------

>MdJAZ7

--------------------------------------------------------------------------------------------------------------------------------------------------------------------------------------------------------------------MRNSNLELH------HPSADSRFPNPNETQQQE-----------------------------------------------------------------------------QQQRLTIFYNGMVCVCD-VTELQ------------------------ARSILF-----L---ANK-EME-------------------------EGVNSLFT---------------------------------PPSGS----AEPPSPNVMS------------------PLCSP------------------------------------VAGMSMKKSLQRFLQKRKH-RTQAT-SPYHH---------------------------------------------------------------------------------------------------------------------------------------------------------------------------------------------------------------------------------------------------------------------------------------------------------------------------------------------------------------------------------------------------------------------------------------

>VvJAZ5

----------------------------------------------------------------------------------------MKRRNCNLELR--------------------------------------------------------------------------------------------------------------------------------LLPPNTDYSSPQHHHNMMMETG-----------------------------------------------------------------RGSPQEQQQQQQLTIFYNGRICVCD-VTELQ------------------------ARAILL-----L---ASR-EME-------------------------EKTRTPTA-----------------------------------SDA----TSPSLHS---------------------QLYSA-------------------------------------TGLSMKRSLQRFLQKRKN-RMEAT-SPYHR---------------------------------------------------------------------------------------------------------------------------------------------------------------------------------------------------------------------------------------------------------------------------------------------------------------------------------------------------------------------------------------------------------------------------------------

>VvJAZ6

----------------------------------------------------------------------------------------MKRSNCNLEVR--------------------------------------------------------------------------------------------------------------------------------LLPPNTDYSSPQHHHNMMMETG-----------------------------------------------------------------RGSPQEQQQRQQLTIFYNGRICVCD-VTELQ------------------------ARAILQ-----L---ASR-EME-------------------------EKTRTPTA-----------------------------------SDA----ISSSLHS---------------------QLYSP-------------------------------------TGLSMKRSLQRFLQKRKN-RMEAT-SPYHR---------------------------------------------------------------------------------------------------------------------------------------------------------------------------------------------------------------------------------------------------------------------------------------------------------------------------------------------------------------------------------------------------------------------------------------

>VvJAZ3

--------------------------------------------------------------------------------------------------------------------------------------------------------------------------------------------------------------------METGRGSPQ--------------------------------------------------------------------------------------------------EQPQQQQLTIFYNGRICVCD-VTELQ------------------------ARAILL-----F---ASR-EME-------------------------EKTRTPTA-----------------------------------SDA----ISPSLHS---------------------QIYSP-------------------------------------TGLSMKRSLQRFLQKRKN-RMEAT-SPYHR---------------------------------------------------------------------------------------------------------------------------------------------------------------------------------------------------------------------------------------------------------------------------------------------------------------------------------------------------------------------------------------------------------------------------------------

>VvJAZ8

-----------------------------------------------------------------------------------------MKRNHFSLYM-----------------------------------------------------------------------------------------------------------------KSRNCNLEL------SLLPSTAYRHKMMMEMGRGSPQ----------------------------------------------------------------------EQQQQQQLTIFYDGRVCVCE-VTELQ------------------------ARAIIL-----T---ASR-EVE-------------------------TKTPTASD-----------------------------------VTM----VSPSLQS---------------------QLYSP-------------------------------------TGLSMKRSLRSFLQKRKN-RIEAT-SPYGH---------------------------------------------------------------------------------------------------------------------------------------------------------------------------------------------------------------------------------------------------------------------------------------------------------------------------------------------------------------------------------------------------------------------------------------

>VvJAZ7

---------------------------------------------------------------------------------------MEFTPNLRKQNN--FP-------------SALQESIKMESN------------------------------------------------------------------------------------KPMNLELPL------FPSTAHTSSIPTMRGGGGGGGS--------------------------------------------------------------------PQEQQQRQQLTIFYNGRICVSD-VTELR------------------------ARAIIL-----A---ASR-EME-------------------------ERKRAPLS--------------------------------------------PSMQS---------------------QLCGP-------------------------------------SGVSMKRSLHRFLQKRKN-RREAM-SPYNH---------------------------------------------------------------------------------------------------------------------------------------------------------------------------------------------------------------------------------------------------------------------------------------------------------------------------------------------------------------------------------------------------------------------------------------

>PpJAZ8

-----------------------------------------------------------------------------------------------------------------------------------------------------------------------------------------------------------------------------------MPTPSPPLETPMG-----------------------------------------------------------------------------DWTLDQQQQITIFYNGQVFVSD-ITELQ------------------------ARAMIL-----L--AAGD-----------------------------MAERGISS----------------------------------VSNA----ALLALQS---------------------QIYGP-------------------------------------PGVSMKRSLQSFLQKRKK-RSQEA-SPYNSLQ-------------------------------------------------------------------------------------------------------------------------------------------------------------------------------------------------------------------------------------------------------------------------------------------------------------------------------------------------------------------------------------------------------------------------------------

>OsJAZ2

---------------------------------------------------------------------------------------------MAEERR-----------------------------------------------------------------------------------------------------------------RDDGGDVEV------ELSLRLRTGDDSTSADPAPATV----------------------------------------------------------------------AAEARRNLTIFYNGRMCAVN-VTELQ------------------------ARTIIS-----MASQGNF-GKQQQ-----------------------QQIQGRDDHHYHQGESSSGGGVSTAAARHCDVAGSSSSHSGSGSGS----ATPPRPALVSPRAGLQAAAAAAP-----TMNQP----------------------------------PAASGLSMKRSLQRFLEKRKT-R--AA-APLYARR-------------------------------------------------------------------------------------------------------------------------------------------------------------------------------------------------------------------------------------------------------------------------------------------------------------------------------------------------------------------------------------------------------------------------------------

>AtJAZ3

--------------------------------------MERDFLGLGSKNSPITVKEETSESSRDSAPNRGMNWSFSNK--------VSASSSQFLSFR--PT-------QEDRHRKSGNYHLPHSGS-------------------------------FMPSSVADVYDSTRKAPYSSVQGVRMFPNSNQHEET-----------------NAVSMSMPG------FQSHHYAPGGRSFMNNNNNSQPLVGVPIMAPPISILPPPGSIV---------------------------------------GTTDIRSSSKPIGSPAQLTIFYAGSVCVYDDISPEK------------------------AKAIML-----L---AGN-G---------------------------SSMPQVFSPPQTHQQVVHHTRASVDSSAMPPSFMPTISYLSPEAGS----STNGLGATKATRGLTSTYHNNQANGSNINCPVP----------------VSC--------STNVMAPTVALPLARKASLARFLEKRKE-RVTSV-SPYCLDKKSST----------------------------------------------------------------------------------------DCRRSMSECISSSLSSAT-----------------------------------------------------------------------------------------------------------------------------------------------------------------------------------------------------------------------------------------------------------------------------------------------------------------------

>AtJAZ4

----------------------------------------MERDFLGLGSKLSPITVKEETNEDSAPSRGMMDWSFSS---------KVGSGPQFLSFG--TS-------QQETRVNTVNDHLLSSAA----------------------------------------MDQNQRTYFSSLQEDRVFPGSSQQDQT-----------------TITVSMSEP------NYINSFINHQHLGGSPIMAPPVSVFPAPT----------------------------------------------------------TIRSSSKPLPPQLTIFYAGSVLVYQDIAPEK------------------------AQAIML-----L---AGN-----------------------------GPHAKPVSQPKPQKLVHHSLPTTDPPTMPPSFLPSISYIVSETRSS----GSNGVTGLGPTKTKASLASTRNNQTAAFSM-AP----------------------------------TVGLPQTRKASLARFLEKRKE-RVINV-SPYYVDNKSSI----------------------------------------------------------------------------------------DCRTLMSECVSCPPAHHLH----------------------------------------------------------------------------------------------------------------------------------------------------------------------------------------------------------------------------------------------------------------------------------------------------------------------

>MdJAZ11

---------------------------------------------------------------------------------------MISVTSQGISVP------------------MSNPYLKNHFT------------------------------------------------------------------------------------TTGQNFSTT------TMKHTAPPSALPVTGGSIAGIT---------------------------------------------------------------EPWNNVKTSGSPSQLTIFYAGTVNVYDDISPEK------------------------AQALML-----L---AGNSCSN-------------------------SSSAAQPKAQAPSAKLAAEDGVPVNQRTNIPPPALSSPLSSVSSHT----GSQSVSGSSSTDELMSARTTGRPTSPVNKMEAP-----------ETANAVRS--------VAATSMIPSAVPQARKASLARFLEKRKE-R-------------------------------------------------------------------------------------------------------------------------------------------------------------------------------------------------------------------------------------------------------------------------------------------------------------------------------------------------------------------------------------------------------------------------------------------------

>MdJAZ18

--------------------------------------------MERDFLGLNSKEPVVVVKEETNNDGGKDPGYGRGGRAHWPFLSQVSAVPQFMSFK--AA-------QDDRTKKMVPDSFLSSVS------------------------AGAFDPCQKQTTCESQKNFNHDRQGGIHFSLTAYPRQRYMHSVHRPHDTKM---------ISVTSQGIS------VPMSNPYLKNHFTTTGQNFSTTTMKHTAPPSALPVTGGSIAGI-----------------------------------------TEPWNNVKTSGSPSQLTIFYAGTVNVYDDISPEK------------------------AQALML-----L---AGNSCSN-------------------------SSSAAQPKAQAPSAKLAAEDGVPVNQRTNIPPPALSSPLSSVSSHT----GSQSVSGSSSTDELMSARTTGRPTSPVNKMEAP-----------ETANAVRS--------VAATSMIPSAVPQARKASLARFLEKRKE-R-------------------------------------------------------------------------------------------------------------------------------------------------------------------------------------------------------------------------------------------------------------------------------------------------------------------------------------------------------------------------------------------------------------------------------------------------

>MdJAZ12

--------------------------------------------MERDFLGLNSKEPVVVVKEETKNDGGKDPGFGRGGGAHSSFQNKVSAXPHFMSFK--AA-------QDDRXXKMVPDSFLFSVS------------------------XDAFGACXKQTTYEAQNYFNHDRQGGTQFSLTAYHTQHDVHPVHRPHDAKM---------ISVTSQGIS------VPMSDPYLKNHFATTGQNFSTXTMKHQIFGGIPVTAPLSALPX---------------------------------XGGSIAGITEPWNNVKNSGSPSQLTIFYAGTVNVYDDISPEK------------------------AQAMML-----L---AGNSCSN-------------------------SSNAAQPKSQVPSAKLAAEDGVPVNQATNIHPPALSSPL-SVSSHT----GAQSASGSTSTDELMAARTSRRPTSPVNKMEAP-----------KTANAAGS--------VAATSMIPSAVPQARKASLVRFLEKRKE-RVMSA-APYNFDKKSP-----------------------------------------------------------------------------------------EHGTPESGGVSFGQQGEQR----------------------------------------------------------------------------------------------------------------------------------------------------------------------------------------------------------------------------------------------------------------------------------------------------------------------

>PpJAZ4

----------------------------------MERDFLGLNSKESVLVVKEEINNDGCKDSGYARGAGGAHWPFLN---------KVSALPHLMPFK--AA-------QDDKTKKMVSESFLSSGF-------------------MPISTADAFDHCQKQAPCEIQNYFNHDRQDGTHFSLTAYPMQHDVHSVHRPHDVKM---------ISVTNQGFS------VPVSNPFFKNPFATTGQNFAATTIKQQLQGIPVTAPYSVLPVS---------------------------------------GSTEPWNNSKNSGSPSQLTIFYAGTVNVYDDISPEK------------------------VQAMML-----L---AGNVSSI-------------------------SSNAAQPKTQAPSAKLAVEDGVPVNQLTNTPPSGLSSPL-SISSHT----GVQSVSGSTNTDELMAPRTSGHPTSPVSKMEPP-----------KIVNAVGS--------VAATSMIPSAVPQARKASLARFLEKRKE-RVMIS-APY------------------------------------------------------------------------------------------------NFSKKSPDSNGVNFTQQGEQQ--------------------------------------------------------------------------------------------------------------------------------------------------------------------------------------------------------------------------------------------------------------------------------------------------------------------

>VvJAZ1

---------------------------------------------MERDFMGLSSKEPLAVVKEEIIEGGKDSAFTKGSGIQWPFSNKISALPHFMSFK--TA-------QEDKSKKVASDSLVTSGF-----------------------------DPSNKCPAGEMQKAVNPDRQGGSFAVTAYPVQHDSHLMNHHPHDVKMFPVSNQ--TISVSMGNP------FFKTHFAAAGQNMVGAALKQQLLGGIPVSAPHGILPSAGSFAG----------------------------------------ITEPWNNFKTSGSPSQLTIFYAGTVNVYDDISPEK------------------------AQAIMF-----L---AGN-GASMAS----------------------RMGQPRAQVQTPASKLAAGDGVLANQPMNISPCSGLSSPISVSSHP----VAQSGSGSTSTEEVLAAKTTGVSATPVTKLDSP-----------------------KMLSVAATPMMPSAVPQARKASLARFLEKRKE-RVMST-APYNISKKSPE----------------------------------------------------------------------------------------CATPGSNGMSYTASSGAGASAGAGALSANKE----------------------------------------------------------------------------------------------------------------------------------------------------------------------------------------------------------------------------------------------------------------------------------------------------------

>MdJAZ13

-----------------------------------------------------------------------MQWSFSN---------KVSALPQFLSFK--AP-------QEGRPRKAVHHNTSAFMT---------------------ISTADAFHSSQKSFSGVIQKNFVLDKQAGNHCGMTVYPVPQFDAHL-----------------VHHSQIPIT------LSSPVLQSHAPVGNNGSTIKLQXLGGVPVLSPVSAHPSTTSVV---------------------------------------GTTDLRNASKSAGAPAQLTIFYAGSVNVYNDISPEK------------------------AQAIML-----L---AGD-GLS------PTHCKS-------------PSVAQVHAPIPGPSLGGDGFXGNQSHIKCSVSALPSPLFATSHGGS----HVGGTFNSTNELAIVKPASPIDR-----SEPSP------------VVSSVGS--------AMTNLIPAVPVPQARKASLARFFEKRKE-RMTST-APYNVSGKSPDRSMPGSQVRPHRIPISKIPSTSFKRFRLSTFLESDSSDSRERERERQRERRMDAAKPRRSDSAGSENGESSYFFTVTDKDDGDMSDTKQHFDAKSDPSGNNDDNIVESDIELDVTDVVEPDNDPPQKMGDLSIEVTEEMQDAAQIEKSKAIDAVSEGMASSCCKLDEAMDLITEAIMLNPASAILKATRASFFVKLNKPNAAIRDANAALEINPDSAKGYKIRGMAKAMLGLWEEAASDLHVASKLDYDEEIGLVLKKVEPNVHKIEEHRRKYERLSKEREIKSAEQEGKRKAEAREWDALSALKDG--------------------------------------------------------MTFD------------------FFY-------------------------SYAI

>MdJAZ14

-----------------------------------------------------------------------MQWSFSN---------KVSALPQFLSFK--AP-------QEGRPRKAVHHNTSAFMT---------------------ISTADAFHSSQKSFSGVIQKNFVLDKQAGNHCGMTVYPVPQFDAHL-----------------VHHSQIPIT------LSSPVLQSHAPVGNNGSTIKLQXLGGVPVLSPVSAHPSTTSVV---------------------------------------GTTDLRNASKSAGAPAQLTIFYAGSVNVYNDISPEK------------------------AQAIML-----L---AGD-GLSPTHCKS-------------------PSVAQVHAPIPGPSLGGDGFFGNQSHIKCSVSALPSPLFATSHGGS----HVGGTFNSTNELAIVKPASPIDR-----SEPSP------------VVSSVGS--------AMTNLIPAVPVPQARKASLARFFEKRKE-RMTST-APYNVSGKSPDRSMPGSQVRPHRIPISKIPSTSFKRFRLSTFLESDSSDSRERERERQRERRMDAAKPRRSDSAGSENGESSYFFTVTDKDDGDMSDTKQHFDAKSDPSGNNDDNIVESDIELDVTDVVEPDNDPPQKMGDLSIEVTEEMQDAAQIEKSKAIDAVSEGMASSCCKLDEAMDLITEAIMLNPASAILKATRASFFVKLNKPNAAIRDANAALEINPDSAKGYKIRGMAKAMLGLWEEAASDLHVASKLDYDEEIGLVLKKVEPNVHKIEEHRRKYERLSKEREIKSAEQEGKRKAEAREWDALSALKDGEVTGIHSARELETKLSAASKASRLAILYFTATWCGPCRFISPLYTSLAGKYKKVVFLKVDIDEARDVAANWNISGVPAFFFVRNGKEVDKMVGADKAALERKIAQHAGSI

>PpJAZ3

------------------------------------MERDFLGLSSKNGNLTVKEEANEEAKNSALPRSSGMQWSFSN---------KVSALPQFLSFK--AP-------QEGGSRKTVHDTSAFMTI-----------------------------------------------STADAFHFSQKPFS-----------------------GVIQQFDAH------SVHHSQDQRIPIGFSSTTKLQPLGGVPVVAPVSLLPSKSSLVG----------------------------------------TADLRNGSKSSGPPAQLTIFYAGSVNVYDDISPEK------------------------AQAIML-----L---AGNGPSPTHCKA--------------------PTIAQVPAPIPRPSAGNGVFRNQAHITSTISGLPSHLSVTSHASSH----SGGAFSSTNELAIVKPVGTSASPIDHSEASKVV----------------------SSVGSAMTNLIPAVPVPQARKASLARFFEKRKE-RMMTT-LPYNVSKKSPE----------------------------------------------------------------------------------------CSTPGSDGLSFSFNSSGSCPPQAIN----------------------------------------------------------------------------------------------------------------------------------------------------------------------------------------------------------------------------------------------------------------------------------------------------------------

>VvJAZ11

----------------------------------------MERDFLCLGSGKVLPSGKEESGDSVPARGLGMQWPFPN---------KVSAIPHFLSFK--AV-------QEDRPRRSVYDRPFASSG------------------LMSVSTADALESNQQPHPGVLQKNLNLEKQVGSQFVMTGYPLQHLDARSVRRAHEV----------GKFPASNQP------NHSISVAFSNPVLQSQIAFSGPSVGSSAVKPQPLGGVPVAAPG------------------------------SYPTICSFIGTTDQKNASKPPGPATQLTIFYAGSVNVFDGISPEQ------------------------AQAIML-----L---AGN-VPPV------------------------SPNTTLPASQGQGPIIMPSGGDGFMGNQAHTSQPGSGLPTLISASS----NVGSQPGGGPNSIDELMAGKTKGALTSTNQPEP------------PNVVTSL--------GSTTPTYIPAVPQARKASLARFLEKRKE-RMMSA-SPYNGSTPASN------------------------------------------------------------------------------------------------VVGFSMNSALC----------------------------------------------------------------------------------------------------------------------------------------------------------------------------------------------------------------------------------------------------------------------------------------------------------------------

>AtJAZ9

----------------------------------------------------------------MERDFLGLSDKQYLS--------NNVKHEVNDDAV-----------EERGLSTKAAREWGKSKV----------------------------------------FATSSFMPSSDFQEAKAFPGAYQWGSV-----------------SAANVFRRC------QFGGAFQNATPLLLGGSVPLPTHP-------------------------------------------------------------SLVPRVASSGSSPQLTIFYGGTISVFNDISPDK------------------------AQAIMLCAGNGL---KGETGDS-------------------------KPVREAER------------------------MCRDTPVAATNAMS----MIESFNAAP-------------------RNMIP------------------------------------SVPQARKASLARFLEKRKE-RLMSA-MPYKKMLL-------------------------------------------------------------------------------------------DLSTGESSGMNYSSTSPT-----------------------------------------------------------------------------------------------------------------------------------------------------------------------------------------------------------------------------------------------------------------------------------------------------------------------

>OsJAZ3

-------------------------------MERDFLGAIWRKEEAAGKPEEHSVSRDADYRGGGGGASAAMQWQFPATK-------VGAASSAFMSFRSSAAAAR----EEDPKEAAVFDRFSLSGF-------------------RPPPRPSPGDAFDGAAAMKQRQFGFNGRQQYAAAAQHGHREQGVDSYGVAAPH------------HFPSPSPSP------RHPVPFGHANPMLRVHSLPNVAGGSPYRNQSFSVGNSVAGSTV----------------------------------------GVYGGPRDLQNPKVTQMTIFYDGLVNVFDNIPVEK------------------------AQELML-----L---ASRASIPSPPSAARKSDSPISAAAKLTVPEALPARQIVVQKPEASVPLVSGVSNPITIVSQAVTLPKSFSSSNDSAGP----KSGGLPLAVTPLSQASPSQPIPVATTNASAIMP-----------------------------------RAVPQARKASLARFLEKRKE-RVSSV-APYPSSKS-------------------------------------------------------------------------------------------PLESSDTIGSPSTPSKSSCTDITPSTNNCEDSLCLGQPRNISFSSQEPPSTKLQI----------------------------------------------------------------------------------------------------------------------------------------------------------------------------------------------------------------------------------------------------------------------------------

>OsJAZ4

---------------------------------------MERDFLGAIGKDEEQRRHAEERKESDYFGAGGGAAAAAMDW-------SFASRAALMSFR--SSSSAAAAAAREETRELAFPHFSALDG-----------------------------AKMQQASHVLARQKSFGAESHGIPQYAAAAAVHGAHRGQPP--------------HVLNGARVI------PASSPFNPNNPMFRVQSSPNLPNAVGAGGGAFKQPPFAMGNAV------------------------------------AGSTVGVYGTRDMPKAKAAQLTIFYAGSVNVFNNVSPEK------------------------AQELMF-----L---ASR-GSL-------------------------PSAPTTVARMPEAHVFPPAKVTVPEVSPTKPMMLQKPQLVSSPVPA----ISKPISVVSQATSLPRSASSSNVDSNVTKSSGPLVVPPTSLPPPAQPETLAT--------TTAAAIMPRAVPQARKASLARFLEKRKE-RVTTV-APYPLAKS-------------------------------------------------------------------------------------------PLESSDTMGSANDNKSSCTDIALSSNRDESLSLGQPRTISFCEESPSTKLQI-------------------------------------------------------------------------------------------------------------------------------------------------------------------------------------------------------------------------------------------------------------------------------------

>PpJAZ10

-----------------------------------------------------------------MNLVPLLRPFSLSPSYLE----KNPLPPCHLSRSSNLPNIPPNKQFSKQFASMSRATVELDFF-------------------GMDQHRDPSSPSKSQFQKFLHRQRSFRGIQNAMYKIKPQVLKSVIASGSVLL-------------NHHQHGSET------PMASRKSFSVPSSPKAEQIPFPSLPVYIPTGTSVSSFMPAPAA------------------------------------------AAAASEKLEETTTPLTIFYNGTVSVFN-VPRDK------------------------AESLLK-----L---ALE-GNS-------------------------AKAAESAL---------------------------------AVDSK----LALHSSD----QQQ--------------LL-DP---------------------------------LDGDLPIARRKSLQRFLEKRKE-RLNSV-SPFASHA-------------------------------------------------------------------------------------------------------------------------------------------------------------------------------------------------------------------------------------------------------------------------------------------------------------------------------------------------------------------------------------------------------------------------------------

>VvJAZ2

------------------------------------------------------------------MSRAAMELDFFGME-------KDGATAKSQFQR--FS-------DRRRSFRGMQSAISKINP---------------------------------------------------------DLLKTVIASGS----------------VSQAPKNNP------PFASRKSFSVPSTPKADQNYLPALPVYSP-----------------------------------------------------------VFRPVSAETTPITIFYNGTVSVFD-VSPEQ------------------------IESIMK-----LALDGST-KTV-------------------------EPADSKLA---------------------------------IPPNE----EQQLLET-----------------------------------------------------------LNGDLPLARRKSLHRFLEKRKE-RLTSV-YPYPPHIQYA-----------------------------------------------------------------------------------------VSDQEKTTMEELFRA--------------------------------------------------------------------------------------------------------------------------------------------------------------------------------------------------------------------------------------------------------------------------------------------------------------------------

>AtJAZ10

-----------------------------------------------------------MSKATIELDFLGLEKKQTNNA-------PKPKFQKFLDRRRSFR-------DIQGAISKIDPEIIKSLL------------------------------------------------------------------------------------ASTGNNSDS------SAKSRSVPSTPREDQPQIPISPVHASLA-----------------------------------------------------------RSSTELVSGTVPMTIFYNGSVSVFQ-VSRNK------------------------AGEIMK-----V---ANEAASK-------------------------KDESSMET-----------------------------DLSVILPTT----LRPKLFGQN---------------------------------------------------------LEGDLPIARRKSLQRFLEKRKE-RLVST-SPYYPTSA------------------------------------------------------------------------------------------------------------------------------------------------------------------------------------------------------------------------------------------------------------------------------------------------------------------------------------------------------------------------------------------------------------------------------------

>OsJAZ5

---------------------------------------------------------------------------------------MSTRAPVELDFL--GLRAAAADADDRHAKSGGSSASSSSS-------------------------------------------------------------------------------------IRGMETSAI------ARIGPHLLRRVIAAAGPPPPPSTAP------------------------------------------------------------VPEEMPGAAAAAAPMTLFYNGSVAVFD-VSHDK------------------------AEAIMR-----M---ATE-----------------------------ATKAKGLA----------------------------------RGNA----IVGNFAK---------------------------------------------------------------EPLTRTKSLQRFLSKRKE-RLTSL-GPYQVGGPAAV----------------------------------------------------------------------------------------GATTSTTTKSFLAKEEEHTAS--------------------------------------------------------------------------------------------------------------------------------------------------------------------------------------------------------------------------------------------------------------------------------------------------------------------

>AtJAZ1

------------------------------------------------------------MSSSMECSEFVGSRRFTGKK-------PSFSQTCSRLSQ--YL-------KENGSFGDLSLGMACKPD----------------------------------------VNGTLGNSRQPTTTMSLFPCE-----------------------ASNMDSMVQ------DVKPTNLFPRQPSFSSSSSSLPKEDVLKM-------------------------------------------------------TQTTRSVKPESQTAPLTIFYAGQVIVFNDFSAEK------------------------AKEVIN-----L---ASK-GTA-------------------------NSLAKNQT--------------------DIRSNIATIANQVPHPRK----TTTQEPIQ--------------------SSPTP----------------------------------LTELPIARRASLHRFLEKRKD-RVTSK-APYQLCDP-------------------------------------------------------------------------------------------AKASSNPQTTGNMSWLGLAAEI-------------------------------------------------------------------------------------------------------------------------------------------------------------------------------------------------------------------------------------------------------------------------------------------------------------------

>AtJAZ2

-----------------------------------------------------------------MSSFSAECWDFSGRK-------PSFSQTCTRLSR--YL-------KEKGSFGDLSLGMTCKPD--------------------------------------------VNGGSRQPTMMNLFPCEAS---------------------GMDSSAGQE------DIKPKTMFPRQSSFSSSSSSGTKEDVQMIK-------------------------------------------------------ETTKSVKPESQSAPLTIFYGGRVMVFDDFSAEK------------------------AKEVID-----L---ANK-GSA-------------------------KSFTCFTA---------------EVNNNHSAYSQKEIASSPNPVCS----PAKTAAQEPIQ-----------------PNPAS---------------------------------LACELPIARRASLHRFLEKRKD-RITSK-APYQIDGSAEA----------------------------------------------------------------------------------------SSKPTNPAWLSSR----------------------------------------------------------------------------------------------------------------------------------------------------------------------------------------------------------------------------------------------------------------------------------------------------------------------------

>MdJAZ1

-----------------------------------------------------------MSSSSETAEVSGQRGMRTAEKP------SNFTQTCSMLCQ--YL-------KEKGSFGDLNLDTACNNM------------------------------------------QQSNGGTPEMFRQKAPPMNFFPFVEN----------------SRNMPTAVR------DFKSMDLFPQQAGFGPSAPTPREEVPMTADS------------------------------------------------------SVKKSAPGEPXKAQMTIFYGGQVIVFNDFPADK------------------------AKEVML-----L---ASK-ESS-------------------------QSHTAPAS---------PPAKTNNAFASHLGKSPVNSSSSVXPSSN----MFPNFGNQAIQEGVK-------------PSPRP---------------------------------VVCDLPIARKASLHRFLEKRKD-RLNTL-APYQTSSPASSP---------------------------------------------------------------------------------------AKPTENKSWLGLAAQQTQ-----------------------------------------------------------------------------------------------------------------------------------------------------------------------------------------------------------------------------------------------------------------------------------------------------------------------

>MdJAZ15

-------------------------------------------------------------MSSSSEKFSGQRGMRTAEKP------SSFTQTCSMLCQ--YL-------KEEGSFGDLNLDMACNNM------------------------------------------QQSNGGAPEMFHQKAPPMNFFPFMET----------------SRNMPTAAR------DFKSMDLFPQQAGFGPSAPTPREEVPMMADS------------------------------------------------------SVKKSAPGEPQKAQMTIFYDGQVIVFNDFPADK------------------------AKEVML-----L---ASK-ESS-------------------------QRHTTPAS---------TPANINNAFASHLGKSPINSSSSVPPSTN----MLPKFDNQAIQEGDK-------------PSPRP---------------------------------IVSDLPLARKASLHRFLEKRKD-RLSTR-APYQTSSPSSGP---------------------------------------------------------------------------------------VKPTENKSWLGLAAQQTQ-----------------------------------------------------------------------------------------------------------------------------------------------------------------------------------------------------------------------------------------------------------------------------------------------------------------------

>PpJAZ1

-----------------------------------------------------------MSSSSETLEVSGQRGLRMAEKP------SSFTQTCSMLCQ--YL-------KEKGSFGDLSLDMACNMQ------------------------------------------QSNGTGTPEMFHQKAPPVNFFPFMEN----------------SRNLPATPG------DFKSMDLFPQQAGFGSSVPRGDVPKMADS--------------------------------------------------------SVKKSVPGEPQKAQMTIFYGGQVIVFDDFPADK------------------------AKEVML-----L---ASK-ESS-------------------------HSQAAQAS---------IPAKSNNVFASHLGKNPMNSSSSVPPSAN----MFPKFGNQVIQEAPK-------------PSPQP---------------------------------IVCDLPIARKASLHRFLEKRKD-RINNK-APYQTSSPAAG-----------------PA---------------------------------------------------------------------KPAEGKS-WLGLAAQPTQ-----------------------------------------------------------------------------------------------------------------------------------------------------------------------------------------------------------------------------------------------------------------------------------------------------------------------

>VvJAZ9

--------------------------------------------------------MSSSSDIADSGRFTGQRAPARGPEK------SSFSQTCSLLSQ--YI-------KEKGTFGDLSLGMTCSLE--------------------------------------GNGTPESLRQTATTTTMNLFPMTERSAG------------------VSGIPARNM------NLKSMNLFPQQAGFGSSVSKDDAPKIVNS--------------------------------------------------------SVKKSGNVEPQTAQMTIFYGGQVIVFNDFPADK------------------------AKEVMR-----L---AGM-GSS-------------------------PVPSTTVK------------------------NPIDAGGMAPSNPN----VVPNFANSLIQERIQ-------------RPAQP---------------------------------VACELPIARKASLHRFLEKRKD-RITAR-APYNISNSPAGP---------------------------------------------------------------------------------------HKPAESKSWLGLAAKSPK-----------------------------------------------------------------------------------------------------------------------------------------------------------------------------------------------------------------------------------------------------------------------------------------------------------------------

>MdJAZ10

---------------------------------------------------------------------------MAAEK-------LNFAQTCNLLSQ--FL-------KEKRTLQVIPPTTMNLLT------------------------------------------------------------------------------------TMEAAASNS------VDQTAQTPSSKPSIDLLPQFTKYPEAALG-------------------------------------------------------------DQQPGSAAQMTIFYGGQVLVFNDLQAEK------------------------AKEIMG-----LATTGSS-KIS-------------------------AGFVKKLG-------------------------------SENQSNV----VAENKSQ---------------------EIKVP--------------------------------------TQARRASLHKFLAKRKE-RVTAV-APYQLNNQRAS----------------------------------------------------------------------------------------SPAKSDEQTSSRAEGQSSKQLELSL----------------------------------------------------------------------------------------------------------------------------------------------------------------------------------------------------------------------------------------------------------------------------------------------------------------

>MdJAZ17

---------------------------------------------------------------------------MAAEK-------LNFSQTCNLLSQ--FL-------REKRTLQVVPPTTMNLLT------------------------------------------------------------------------------------TMEAADSNP------AHQAAQTPSSKPNMELFQHFTKGPEAVLG-------------------------------------------------------------DQQPGSAAPMTIFFGGQVLVFNDLQAEK------------------------AKEIMD-----LATIGSS-KSS-------------------------GRFVEKLA---------------------------SGNQSNVVAKN----NYQEIQK---------------------VQPQA---------------------------------AASDLPIARRASLHKFLAKRKE-RVAAI-APYQLNNQRAS----------------------------------------------------------------------------------------SPAKSDEQTSSRAEDQSSKQLELRL----------------------------------------------------------------------------------------------------------------------------------------------------------------------------------------------------------------------------------------------------------------------------------------------------------------

>PpJAZ5

----------------------------------------------------------------------------MAEK-------VNFAQTCNLLSQ--YL-------KEKRSLQVPTTMDL----------------------------------------------------------------------------------------LTNMETGPA------AETPSSKPSIDLFPQFAKNPEA-------------------------------------------------------------------VFSNQPGSAQMTIFYGGQVLVFNDLQAEK------------------------AREIMN-----FATKGSS-KIS-------------------------SGFVSNGI-------------------DKFGSASVTKMVASEPNIA----ANSQDIQ---------------------KVHSQ--------------------------------VIGSDLPIARRASLHKFLAKRKE-RVAAI-APYQVNHQRA-----------------------------------------------------------------------------------------SPSKSEEEMSSRDQVEGQCSKQLELRLY-------------------------------------------------------------------------------------------------------------------------------------------------------------------------------------------------------------------------------------------------------------------------------------------------------------

>VvJAZ4

-----------------------------------------------------------MSNSPEFSDFAGRKSGKLPDR-------SNFSQTCNLLSQ--FL-------KEKGRFGDLSLGMAGKSE-------------------------------------------TKGRPESFKSSTMSFDLLNKDKSSEA---------------SGQNVGGSS------NLKSSDFYPQFAGFGSLASIDEAINMADF----------------------------------------------------------RKSATTESETSQMTIFYAGQVLVFNDFPAEK------------------------AREVML-----L---AAK-GT--------------------------PQNTSGFLSTSGPEKINTGSSTAPSPSIPASPATTPNPQALSSGTF----SIPASPAATP------------------NPQAP---------------------------------LGSELPIARRNSLHRFLEKRKD-RVNSK-APYQVNNPSRP----------------------------------------------------------------------------------------SPKPEEDTNPKLNKDEGQSSKQLDLRL--------------------------------------------------------------------------------------------------------------------------------------------------------------------------------------------------------------------------------------------------------------------------------------------------------------

>AtJAZ5

------------------------------------------------------------------MSSSNENAKAQAPEK------SDFTRRCSLLSR--YL-------KEKGSFGNIDLGLYRKPD------------------------------------------------------------------------------------SSLALPGKF------DPPGKQNAMHKAGHSKGEPSTSSGGKVK---------------------------------------------------------DVADLSESQPGSSQLTIFFGGKVLVYNEFPVDK------------------------AKEIME-----V---AKQ-AKP-------------------------VTEINIQT-------------------PINDENNNNKSSMVLPDLN----EPTDNNHLTKEQQQ--------------QQEQN----------------------------------QIVERIARRASLHRFFAKRKD-RAVAR-APYQVNQNAGH-------------HRYPPKP--------------------------------------------------------------------EIVTGQPLEAGQSSQRPPDNAIGQTMAHIKSDGDKDDIMKIEEGQSSKDLDLRL-----------------------------------------------------------------------------------------------------------------------------------------------------------------------------------------------------------------------------------------------------------------------------------

>AtJAZ6

-------------------------------------------------------------------MSTGQAPEK-----------SNFSQRCSLLSR--YL-------KEKGSFGNINMGLARK--------------------------------------------------------------------------------------SDLELAGKF------DLKGQQNVIKKVETSETRPFKLIQKFSIGEASTSTEDKAIYID-----------------------------------------LSEPAKVAPESGNSQLTIFFGGKVMVFNEFPEDK------------------------AKEIME-----V---AKE-----------------------------ANHVAVDS-------------------KNSQSHMNLDKSNVVIPDL----NEPTSSGNNEDQET--------------GQQHQ-----------------------------------VVERIARRASLHRFFAKRKD-RAVAR-APYQVNQHG------------------------------------------------------------------------------------------SHLPPKPEMVAPSIKSGQSSQHIATPPKPKAHNHMPMEVDKKEGQSSKNLELKL-----------------------------------------------------------------------------------------------------------------------------------------------------------------------------------------------------------------------------------------------------------------------------------

>OsJAZ6

--------------------------------------------------------------------MASAKSGERGS--------SSFAMACSLLSR--YV-------RQN---GAAAGELGLG--------------------------------------------------------------------------------------IRGEADANK------GKETMELFPQNSGFGSEAAAVK-----------------------------------------------------------------ETPDAREQEKRQLTIFYGGKVLVFDDFPAEK------------------------AKDLMQ-----M---ASK-----------------------------SSSTAQNC-------------VLLPSSATATVADNTKVSAVPAPAS----ALPVAQA---------------------NAPKP------------------------------VRPNAADLPQARKASLHRFLEKRKD-RLQAK-APYQGSPSDAS-------------PVKK-----------------------------------------------------------------------ELQESQP-WLGLGPQVAAPDLSLRQESSQ------------------------------------------------------------------------------------------------------------------------------------------------------------------------------------------------------------------------------------------------------------------------------------------------------------

>OsJAZ7

-------------------------------------------------------------------MAASARPVGVGGERA-----TSFAMACSLLSR--YV----------RQNGAAAAELGLGIR-------------------------------------------------GEGEAPRAAPATM----------------------SLLPGEAER------KKETMELFPQSAGFGQQDAITA--------------------------------------------------------------DSAADAREQEPEKRQLTIFYGGKVLVFNDFPADK------------------------AKGLMQ-----L---ASK-GSP-------------------------VAPQNAAA-----------------PAPAAVTDNTKAPMAVPAPVS----SLPTAQA---------------------DAQKP------------------------------ARANASDMPIARKASLHRFLEKRKD-RLNAK-TPYQASPSDAT-----------------PVK--------------------------------------------------------------------KEPESQP-WLGLGPNAVVKPIERGQ----------------------------------------------------------------------------------------------------------------------------------------------------------------------------------------------------------------------------------------------------------------------------------------------------------------

>OsJAZ8

-----------------------------------------------------------------MAGRATATATAAGKDR------SSFAVTCSLLSQ--FL-------KEKKGGGGGLQGLGLGLR----------------------------------------PAPAAPPAAGAGGAFRPPPT------------------------TMNLLSGLD------APAVEVEPNTAETAADELPLIKAPADQQS--------------------------------------------------------DESASEAAGEKAQQLTIFYGGKVVVFENFPSTK------------------------VKDLLQ-----I---VSTGDGV-------------------------DKNTGTAA-------------------------------TQSLPRP----AHNSLPD---------------------------------------------------------------LPIARRNSLHRFLEKRKG-RMNAN-APYQANCTAAPS---------------------------------------------------------------------------------------KQANGDKSWLGFGQEMTIKQEI-------------------------------------------------------------------------------------------------------------------------------------------------------------------------------------------------------------------------------------------------------------------------------------------------------------------

>OsJAZ11

----------------------------------------------------------MAGSSEQQLVANAAATTVAGNG-------SRFAVTCGLLRQ--YM-------KEHSGSNGGGGFLPAVTA------------------------------------------------------------------------------------MSLMTGGAD------AEEEAPEVRKTMELFPQQAGTL---------------------------------------------------------------KDTQERKEITEKAQLTIFYGGSVVVFDDFPAEK------------------------AGELMK-----L---AGS-RDS-------------------------TAAAAVSD---------------------------------AGAAA----GQPCLPD---------------------------------------------------------------MPIARKVSLQRFLEKRKN-RIVVA-EPLPESE--------------------------------------------------------------------------------------------KKEAESSKRAKKDDGGASWLQVNPTLSL-------------------------------------------------------------------------------------------------------------------------------------------------------------------------------------------------------------------------------------------------------------------------------------------------------------

>OsJAZ12

-------------------------------------------------------------------------MAAAGSS-------SRFAVTCGLLSQ--YM-------RERQQPQPPVTVLE----------------------------------------------------------------------------------------AVAEEEEEE------DARTMQLFPPRAAAADGVATPS------------------------------------------------------------------------AGTAPLTIFYDGRMVVVDDVPVEK------------------------AAELMR-----L---AGS-ACS------------------------------------------------------------------PPQPA----HAAALPE---------------------------------------------------------------MPIARKASLQRFLQKRKH-RITTTSEPYKKAAVASP----------------------------------------------------------------------------------------APEKSFA-VAPVKDEPATWLGL-------------------------------------------------------------------------------------------------------------------------------------------------------------------------------------------------------------------------------------------------------------------------------------------------------------------

>OsJAZ10

-------------------------------------------------------------------------MAMEGKS-------RRFAVACGVLSQ--YV-------RAEQKMAAAAGAAPARAV------------------------------------------------------------------------------------TTLSLMPGA------EVVVEEEERREVGEEEAGPATA-------------------------------------------------------------------------PAAPLTIFYGGRMVVFEDFPADK------------------------AAEVMR-----M---ASS-GMA-----------------------------------------------------------------AAPAQR----EGAALAD---------------------------------------------------------------MPIMRKASLQRFFAKRKD-RLAAT-TPYARPSPAET---------------KASEP--------------------------------------------------------------------EEKKTPTSWLDLAASASAAARRDSLTIAL------------------------------------------------------------------------------------------------------------------------------------------------------------------------------------------------------------------------------------------------------------------------------------------------------------

>OsJAZ13

------------------------------------------------------------------------MAAEAAAT-------SRFAAACGALSQ--YV------------RAADNVHRARTAA------------------------------------------------------------------------------------AAAAVRPLP------LMPGADVAGDEREEEGGGAAAS------------------------------------------------------------------------SAAAQMTIFYGGRVLVLDECPADR------------------------AAALLR-----L---AAS-----------------------------SRGVPRDD--------------------------------LASTAA----AAGESAD---------------------------------------------------------------LPVARKASLQRFMEKRKG-RLAARGQPYRRHDAAAA------------------------------------------------------------------------------------------ARGDHLALAL-----------------------------------------------------------------------------------------------------------------------------------------------------------------------------------------------------------------------------------------------------------------------------------------------------------------------------

>OsJAZ14

-------------------------------------------------------------------MAVSDHHCGGGGRS------WRFAVACGVLSR-----------------------------------------------------------------------------------------------------------------CVKAEAAAA------ANGRHRHHPTMLLMPGADVEPD-------------------------------------------------------------------VREEAAAAAQLKIMYGGRMLVFDDFFPAG------------------------GAVVEL-----V---RAA-----------------------------ARAGQDVR-------------------------------RAGAARR----RVGDSRG-----------------------------------------------------------LDAGLPVVRKVSLQRFVEKRRRMRVYH--ILYTDKSSHHVPGPGRYRSWQCRIIIAAVAGAGGFVVACGVLSRCV-----------------------------------------------------KAEAAAAAANGRRHHHHHHTTMLLMPGADVEPDVREEAAAAAQLKIMYGGRMLVFDDFFPAGGAVVELVRAAARAGRDDDGARARRRPAGGEEGVAAAVRGEEKSQAARGDGAVHTRHSPPMLPARTPGSGRTDDAAFY----------------------------------------------------------------------------------------------------------------------------------------------------------------------------------------------

>OsJAZ9

--------------------------------------------------------------------MASTDPMT-----------RRFAVACGVLSQ--YV-------------------------------------------------------------------------------------------------------------KANSSQPST------AAPVAQGVSGLMAAAAAAAAAP--------------------------------------------------------------VVQEPGCEVDGGGQQFTIFYAGKVVVIDRCTPAM------------------------AAELMR-----F---ASA----------------------------------------------------------------------AQGGG----GAPEAPP-----------------------------------------------------------ALVDMPIARKASLKRFLAKRKATPASAR-SSYVVRAAAAE----------------------------------------------------------------------------------------EEQPPAKKAKAAVERREDWLALGSLGHMHSR----------------------------------------------------------------------------------------------------------------------------------------------------------------------------------------------------------------------------------------------------------------------------------------------------------

>PP00103G00080

-------------------MCNPCRRYYPAIEAPVVSLNLFSSIPISSYTDDLDTTAYAFDLGSLNRPSWVNDHIAAGGRQADLRNEDNSGVSIKTSVK-----------KDIVYDCHSANSVSSGLFCFCKLFISYSKCQHLHWYTAMTQCSCVLDVKQKSFLSRFELDISQQRTWKAGSEYDGHQQALRNIPIRASLLKGEAQTTSVHQTNLSGNKPFP------ILHQYSSSNHSAGFGSREPLCSVMKNSYGVKFRSLQVSENLPH-----------------------QQNLQHPTHDANMAAADALATTPATRRLPLAAQLTIFYADMVNVYEDVPYDK------------------------AQAIML-----L---ASR-ESH-------------------------SNYLNPLVSYGASAHPGVCSFGAMSQPRLRTSSPAPSACAGRPPPG----DIPIAIR---------------------RTPTR----------------------------------TIELPQARKASLARFLERRRD-RARTR--PYAQRNEALR-----------RIRDASPSP--------------------------------------------------------------------SLSTGRP-PARPLSPPATENKNGESSNASGASTTDNEANSPPPIQPSQSMSE----DNQMPRITQTPNVLPSPIAVLSECEAVVR----------------------------------------------------------------------------------------------------------------------------------------------------------------------------------------------------------------------------------------------------

>Pp3c5_11730

--------------MTREPVAIDLINLGGIAERGVGGLMRSSSIECDERVSRGRLVRPVPSMLNHPSRSQSVSRMSPGPDGLRDRNINYFTQPGPFRPPWMSNHVAAGRVLTDCRSHCNFGMAAETSAERNMVSEDHLTGSQSRGDHSAPASPSHHIRLSSSSGYELDFTQQHTRKYASIFTSPAHALRNMPASTSSLREEAQAMSTHQSNLTAAEPSSVPRQHPSSSHSAGFGSPVPFYSRMKDNTGAESGSALGSQNPPRQPELQHPV-------------------------------KDTKMAAAHGLVRSATVGELPRVAQLTIFYAGMVNVYDHVPYEK------------------------AQAIML-----L---AGR-ESY-------------------------PNYESLLGGCSATESPWICSPGAINLQASRGGSPAPLSSADLPPPG----VVPMAIR---------------------PTPTT---------------------------------TAVELPQARKASLARFLERRRD-RVRTG--PYVPRNEEAR-----------RIRENPPSP--------------------------------------------------------------------SVSSARP-PTRP-SSPVPGHNNTAPPNAGGATTSTSQVNAPPPTPSRESSSEVLDPSPGRPQGENGEGMQPAPPGVSSVGEGSVAMS--------------------------------------------------------------------------------------------------------------------------------------------------------------------------------------------------------------------------------------------------

>Pp3c16_13490

-------------------------------------------------------------MGIEGATQPQVRPIPSASG-------SHLAEAPAMSRMPPLV-------EGVCSRDSSNNNMDRDTS-------------------------------------------FFARPGVTRSPWMNMPTITKTLPAD----------------SARSEDNSV------MTVDDVFLKKEVMQSRFEDSALSQQARKVASMNVNHLRQAASH----------------------------------------VFARAPGSSKQPPTAQLTIFYAGMVNVFDDVPLDK------------------------AQAIML-----L---AGT-DST-------------------------CSSNHMNL----PGASVRPFPTRMSQPSSRVGSPAPQMTTSSAGTA----ALPGAPRAVNRQAL--------------TAATT--------------------------------GLIVELPQARKASLARFLEKRKD-RVRK--GPYTDSRNEEA------------ARDDESRR--------------------------------------------------------------------SGRQNSPCPSDPKGKIPARSLSPPATKNQGNAPFPGGASTSFSELNSPPQTPPRKSMSDDRSHETLEKSKESESRIEAMVPSGEKLSSPSRNGTGSRTEPMDEHSS-------------------------------------------------------------------------------------------------------------------------------------------------------------------------------------------------------------------------------

>Pp3c25_6300

-------------------------------------------MSKMSVIPEQLSSRDYDDDNNNNFNNSSSSSSSSRRRDLDNSFYSRPGLSQSPWLNKHSMAKRVPADPARIEENSVTAVIDTSLT-----------------------KETMEGSHPTMNSRNPYEDFALVQQAGTATSMHIDLLQSFKSNVRTA--------------PHIGNAQLS------AHRSDPSASEPSSLPQQHPSSNSARFVGLAPFFSRMRGNSRVE-------------------------NESLPQLQHQQATAHAFSRPPGTGKLIESAQLSIFYAGMVNVYDAVPIEK------------------------AQAIML-----L---AGT-RSA-------------------------WSSPNHMNLPGAPGHPFPASINQPPFRTRSPGPQMISGYAGTTALS----GVPKATNRQV------------------PTAET--------------------------------GFIVELPQARKASLARFLDKRKD-RVRKG--PYNDPRNENL-------------ARYDELR--------------------------------------------------------------------TSRENSPCPSDSKGKAIALSLSPSATRNQRNSPFPGDPGHASSASASSSEPTSRETME-------------------------------------------------------------------------------------------------------------------------------------------------------------------------------------------------------------------------------------------------------------------------------

>PP00442G00070

-------------------------MAGEPVAVDYMGVGGAPTSSPDGTEERVRLVRPIPTVPDNRAGNFRISPAPEGGRDNSPFVHHGLRQQAWNRVQ---VAARFQLENENSNKVVVCPKKEKETTDGPLNGHPQFSSQWDNSAPTSPSHHLGSSANRYDAFLRMHQSEKVTTTYGNHQALKGMQNGGGRTSPFRDEAQRGAQPS-----DHATQPHPP------SRQGGCGAPTPFCARSNSGVVDSVAAMAGKKLPFQHPVQSGSL------------------------------------------PRPGGLGKQPRTAQLTIFYAGMVNVYDDVPFDK------------------------AQAITL-----L---AGS-RNTWSS----------------------NFMNPPQAGSAASGRTFSTPTAVPPSTPSTPGSPAPQASTTSAPAP----PRPSLSGVVFS-----------------NVRQP-------------------------------PIHNFELPQARKASLARFLEKRKD-RVKKV-IVKEEEDASPP----------------------------------------------------------------------------------------RGNSEGPSGGKPPSRSPSPSPAVSRSMDYLSSPGRVEHQAGTSSCSERSSPSNPRPPQTPPSQSQSEERSAGTTKRRGFGLAHEPNKRARNGKSQCRLSGESAADHIVEDGSFPHR---------------------------------------------------------------------------------------------------------------------------------------------------------------------------------------------------------------------

>Pp3c5_11800

------------------------------------------------------MEKETTDGPLNGHPQFSSQWDNSAPT-------SPSHHLGSSANR--YDAFLRMHQSEKVTTTYGNHQALKGMQ------------------------------------------------NGGGRTSPFRDEAQRGAQPS----------------DHATQPHPP------SRQGGCGAPTPFCARSNSGVVDSVAAMAGKKLPFQHPVQSGSL------------------------------------------PRPGGLGKQPRTAQLTIFYAGMVNVYDDVPFDK------------------------AQAITL-----L---AGS-RNTWSS----------------------NFMNPPQAGSAASGRTFSTPTAVPPSTPSTPGSPAPQASTTSAPAP----PRPSLSGVVFS-----------------NVRQP-------------------------------PIHNFELPQARKASLARFLEKRKD-RVKKV-IVKEEEDASPP----------------------------------------------------------------------------------------RGNSEGPSGGKPPSRSPSPSPAVSRSMDYLSSPGRVEHQAGTSSCSERSSPSNPRPPQTPPSQSQSEERSAGTTKRRGFGLAHEPNKRARNGKSQCRLSGESAADHIVEDGSFPHR---------------------------------------------------------------------------------------------------------------------------------------------------------------------------------------------------------------------

>Pp3c6_23650

-------------------MVDWRAMAREPVAVDYMGVGGAPSSISGGHEERVSLVRPIPSVPNNGGGHFRRSPAPEGGPHS-----SPFEQHDPDQKSWKRASFLARFQSEEDNSRRVIICPNKERESTDGHLNDHPQSSSQWNNSESKSHTHHPQSQKVAAMYASHHHLLRGIQNGDAWTSPLKDEVQRGAQPL----------------DHAMQPHLS------SNQGRFESPMPFGGHRLGSVESGAAVARKKPPFQYPFQAGNL-------------------------------------------PRPGGPCKQPRTAQLTIFYAGMVNLYDDVPVDK------------------------AQAIML-----F---AGS-EST-------------------------WSSNLMDPPQAGSVASGRTFSAPTTVPLSTPGPPAPQALTTSASGP----PSSVLPGMVFS-----------------NLRQP-------------------------------STTNVELPQARKASLARFLEKRKD-RVKKD--PVKEGDATPF-----------------------------------------------------------------------------------------GNSPDPSIGKPPTWSPSPSPSVSRCMDHGSSPGRLEHQSGTSSGNEQNSPCNSRPPQSPPSQGVAE----------------------------------------------------------------------------------------------------------------------------------------------------------------------------------------------------------------------------------------------------------------------

>Pp3c25_6330

-------------------MARDAVAVDFMGIGERRGGVSYDDNEERVRLVRPVPTVPEDRQGQSRTWPGAEEWRNPGDRDASPFTQRLPALHRSPWSR-LPIASRFQSDEENSSKAMVSPKREKDLKEGLPTGHQQSFSAWDNSTPGNPSYHPTGSSSRYEEFVRMQQHLQARKAAVMHADHQQAPRCVQNAGARTSPFRDEAQR------GAQPSDRLG------PEHLTTQPHAPSNQSGLGSPGPFHGRGSGTAFTGRRIPFQAGN-------------------------------------LSNHVVRRGGTGAKPRTAQLTIFYAGMVNVYDDVPFDK------------------------AQAIML-----L---AGS-GST-------------------------WSSNNMGHRGSGPARPFSAPTAVPQPTPSTPGSPAPQGSTTSAAGS----LRPVIPGVMFS-----------------SVRQP-------------------------------PVANVELPQARKASLARFLEKRKD-RVRK--VPVKAEGETSPS---------------------------------------------------------------------------------------RDKSPTPSCGNAPSRSSSPCPVGQERGSSPACGQGHQPGASSCSEPNSPTIPSQTPPTESASEEKSSIGTPKRKDVEMEQESHKRARIGRSPPRVAGRSTNDVMEQHES----------------------------------------------------------------------------------------------------------------------------------------------------------------------------------------------------------------------------

>AtJAZ11

-----------------------------------------------------------------MAEVNGDFPVPSFAD-------GTGSVSAGLDLL-----------VERSIHEARSTEPDASTQ-------------------------------------------LTIIFGGSCRVFNGVPAQKVQEIIR----------------IAFAGKQTK------NVTGINPALNRALSFSTVADLPIARRRSLQRFLEKRRD-----------------------------------------------RSTKPDGSMILPSQLTIIFGGSFSVFDGIPAEK------------------------VQEILH-----I---AAA-----------------------------AKATETIN-----------------------------LTSINPALK----RAISFSNAS-------------------TVACV---------------------------------STADVPIARRRSLQRFFEKRRH-RFVHT-KPYSATTSEA-----------------------------------------------------------------------------------------DKNETSPIVT-------------------------------------------------------------------------------------------------------------------------------------------------------------------------------------------------------------------------------------------------------------------------------------------------------------------------------

>AtJAZ12

---------------------------------------------------------------------------------------------------------------MTKVKDEPRASVEGGCG------------------------------------------------------------------------------------VADGDGGAA------EIGGTGSVEKSINEVRSTEIQT--------------------------------------------------------------------AEPTVPPNQLTIFFGGSVTVFDGLPSEK------------------------VQEILR-----I---AAK-AME-------------------------TKNSTSIS-------------PVSSPALNRAPSFSSTSNVASPAAQ----PFPIQPI---------------------SFCRS----------------------------------TADLPIARRHSLQRFLEKRRD-RLVNK-NPYPTSDFKKT----------------------------------------------------------------------------------------DVPTGNVSIKEEFPTA-------------------------------------------------------------------------------------------------------------------------------------------------------------------------------------------------------------------------------------------------------------------------------------------------------------------------

>MdJAZ2

------------------------------------------------------------------------------ME-------EKAEVGCDXKVK-----------------------------------------------------------------------------------------------------------------LSEMEEEMV------AQNKPNQTEDDLQNTRKVPSAL--------------------------------------------------------------------NITGPAPAQLTIFYAGSVSVFDAITAEK------------------------VRELML-----I---AAAAAAD-------------------------KKTSDVKN----------SATSCPPSPLIRTGSSTLQXSSTAPGSP----VVQPXPEQN-------------------SSICK---------------------------------LQAEFPIARRHSLQRFLEKRRD-RMVSN-SPYPTSPATPK-------------DDNAKTI--------------------------------------------------------------------QSDNASP-GVGCFKQSAMVQEETQPMHWSIGFRQLGTVAEPQMKVPAGTLYCVRPESTGPRVFSTPHRIAFDFLQFFLSARFHVLCGRAYCNPADIT----------------------------------------------------------------------------------------------------------------------------------------------------------------------------------------------------------------------------------------

>MdJAZ9

---------------------------------------------------------------------------------------MEEKAEVGCDLK-----------------------------------------------------------------------------------------------------------------LSEMEKEMV------AQNKPNQTEDDLQNIRKGPSAL--------------------------------------------------------------------NITSPAPAQLTIFYAGSVSVFDAITAEK------------------------VRELML-----IAAAAAD-KKT-------------------------SDVKNTAT--------------SGPPSPLVRTGSSSLQNSSAPGSP----VVQPYPDQK-------------------SSTSK---------------------------------LEAGFPIARRHSLQRFLEKRRD-RLVSN-SPYPTSPATPM-------------DDSSKTN--------------------------------------------------------------------PSNNASP-GVGCFKQSAMVQEETQPSSVA------------------------------------------------------------------------------------------------------------------------------------------------------------------------------------------------------------------------------------------------------------------------------------------------------------

>PpJAZ2

-------------------------------------------------------------------------------------------------------------MEEKSQAGDVKNTPEMEEK------------------------------------------------------------------------------------VVGQSQPMK------TEEEAAKEKDPSSSHDLPNNSN--------------------------------------------------------------------TTRKIMPAQLTIFYAGSVSVFDAVTAEK------------------------VRELML-----I---AAA-DAA-------------------------NKTADVKN------------GGTSGPPSPLVCTGSSSLQNSAPGSP----VVQPYPDQK-------------------SSICK---------------------------------LQAEFPIARRHSLQRFLEKRRD-RLVSK-NPYPTSPATQL-------------DDDAKIN--------------------------------------------------------------------LSNNASP-GLGCFKQSAMVKEEMQ--------------------------------------------------------------------PSSATAYPA------------------------------------------------------------------------------------------------------------------------------------------------------------------------------------------------------------------------------------

>OsJAZ1

---------------------------------------------------------------------------------------MDLLEKKNIKKG--GE-------VEEEVARKGEERKEEEVV------------------------------------------------------------------------------------VEEKSHQQQ------QQQGEEELVGLSLAGGRPKVFP-------------------------------------------------------------------MSSPPPNPSQLTIFYGGSVCVYDSVPPEK------------------------AQAIML-----I--AAAAAAAA-------------------------SATKSNAAIAVKPPVMPAANATQAAVSPVLTRSLSLQSTSVATGQP----QVAADPS---------------------SICKL----------------------------------QADLPIARRHSLQRFLEKRRDSRLVSK-APYPTKSSEGM----------------------------------------------------------------------------------------EASGMEVTAEGKAQ---------------------------------------------------------------------------------------------------------------------------------------------------------------------------------------------------------------------------------------------------------------------------------------------------------------------------

>MdJAZ16

----------------------------------------------MITKDYFMKTENNNSSDIQEFFXDQKKKNRAIISNAAGFDGEKGASTPNDCQQ-----------ENSSLKPSNDMSWLFRKY---------------------------------------LFTRSHSNSTGKSNTEAESESEIVKRH------------------SSPGPSVPP------MLLGSNDFRGDRLSLLERKLLPGLRC-------------------------------------------------------------GDQSNKTSTTEQLTIFYNGIVHVYDDIPADK------------------------AKEILC-----L---ASE-----------------------------NSSSKPLI----------------------------RERLKKAPPP----LRPQLSV------------------------SK---------------------------------LRAGXPMARRHSLQCFLEKRRG-RIINK-YPYALHPG-------------------------------------------------------------------------------------------KQEDNEAFINNQSNENHKISLSPFPSRLGYFYPKLFNQGC-------------------------------------------------------------------------------------------------------------------------------------------------------------------------------------------------------------------------------------------------------------------------------------------------

>PpJAZ6

----------------------------------------------MACSTPIIIKDFFMKTDQELYGEDKNKSSSGFEKVAR----TPDDQQRDISLR--SM-------DEE--NHMSQSVWFFRKY-------------------------------------------LLTRSQNNVAKTSMEAEFESEIIKG----------------HSSPRPLPP------KFLGSNNFPGHRLSLLEQQLLP---------------------------------------------------------------GLRCDDQSNRTSEQLTIFYDGIINVYDNIPADK------------------------AQAIMR-----L---ASE-----------------------------NSSVKPLV-----------------------------AESFKTDRQ----KPPLKPK---------------------SLSVS--------------------------------KIRAGLPMARRYSLQCFLEKRRD-RNINN-SPYALHSKKQE----------------------------------------------------------------------------------------DNYEATV-NNESNESDKLSLLPFPSRLGYFYPRLVNQGSC-------------------------------------------------------------------------------------------------------------------------------------------------------------------------------------------------------------------------------------------------------------------------------------------------

>VvJAZ10

----------------------------------------------------------------MEKVVMNEHQTKSGLKQT-----NDMSRTISMFRK--YL-------ISKTQSDGGKTNVDESET------------------------------------------------------------------------------------IKRSPLRPL------MAPKFGGGSPRLSSLLEQELLPGLRCDN---------------------------------------------------------TMSDSSDDKFPGAQLTIFYAGTINVYDHITMDK------------------------VQTILH-----FARESSS-----------------------------PTNSEAMI-------------------------PKKDPTIAPSHPS----GLPSFCR-----------------------------------------------------------LQADFPIARKSSLQRFLEKRRD-RITSR-SPYASSSTKRK-------------ENEQKID--------------------------------------------------------------------NYPKEKH-CFSPSAFPSQLGYFFPSIKY-------------------------------------------------------------------------------------------------------------------------------------------------------------------------------------------------------------------------------------------------------------------------------------------------------------

>MdML2

---------------------------------------------------------------------------MYGHS-------QDMTMPNPIPAC-----------DDDDAGAADSIDNAHIQY------------------------------------------------------------------------------------DSHTLEDGG------IVVVEDGSSDGVYVQGGSASSE------------------------------------------------------------------LRGQPYDGSSQLTLSFRGQVFVFDAVTPEK------------------------VQAVLL-----L--LGGN-ELS-------------------------PNAQGTEL--------------------------------ASQNPR----ATEDFP----------------------RCSQP----------------------------------------HRAASLFRFRQKRKERCFDKK-VRYGVRQEVAL----------------------------------------------------------------------------------------RWCSLYXCMCLLN----------------------------------------------------------------------------------------------------------------------------------------------------------------------------------------------------------------------------------------------------------------------------------------------------------------------------

>PpZML5

---------------------------------------------------------------------------MYGHS-------EPMTMSNPIPAG-----------GDDDAAGPGVDSIDNAHIHYEP--------------------------------------------------------------------------------HTLEDGGGV------VAVVEDVSSDPVYDVGSSEMRA---------------------------------------------------------------------QPYDGSSQLTLSFRGQVFVFDAVTPEK------------------------VQAVLL-----L---LGGSELS-------------------------SGPQGAEL--------------------------------ASQNQR----GTEDFPI---------------------RCSQP----------------------------------------HRAASLSRFRQKRKERCFDKK-VRYSVRQEVAL-------------RMQRNKG--------------------------------------------------------------------QFSSSKK-S----DGDYSWGNGQESGQDD----SHAETSCKHCGISSKSTPMMRRGPSGPRSLCNACGLFWANRGTLRELSK-------RTQDHSVTPAEQ--------------------G------EADTKDLNSVTAI-------------------------------------DAHNSLVPFSNGDSSALVAEQ----------------------------------------------------------------------------------------------------------------------------------

>VvZML3

---------------------------------------------------------------------------MYGHP-------QHMSMHNQIAGD--DD-------DGAASESIDNPHVHYDAH------------------------------------------------------------------------------------VLQDGVVPG------MEVAGDVPSDAVYVADGSEVAL---------------------------------------------------------------------QPSDATNQLTLSFRGQVYVFDSVTHEK------------------------VRSVLL-----L---LGT-----------------------------PELSSIAH----------------------------NMEIVPQNQR----ALTDFPG---------------------PYNQP----------------------------------------HRAASLNRFRQKRKERCFDKK-IRYNVRQEVAL-------------RMQRNKG--------------------------------------------------------------------QFSSSKK-S----EGTFSWDSVQDSGQDE----SPPETLCTHCGTSSKSTPMMRRGPTGPRSLCNACGLFWANRGSLRDLSK-------KNQDHSLNQIER--------------------G------DGEANDSDCGTSG-------------------------------------YINNNHVAFSNSDNPALIPEQ----------------------------------------------------------------------------------------------------------------------------------

>PpZML1

-----------------------------------------------------------------------------------------MSESNHQNSM--YG-------SGGAPQSNQVEEQEDDVE------------------------------------------------------------------------------------ESIDNPHIR------FEDSSAIPPNPLYLTSSEYPPA--------------------------------------------------------------------AATNGGSDQLTLSFQGEVYVFDEVSPDK------------------------VQAVLL-----L---LGGYEIP-------------------------SGIPSMGP--------------------------------VPLNQQ----GMNDLPV---------------------KPIQP----------------------------------------QRAASLSRFREKRKERCFDKK-IRYTVRKEVAL-------------RMQRKKG--------------------------------------------------------------------QFTSSKA-SSDD-GGPASSGATQGSGQDE----SMQETSCMHCGISSKSTPMMRRGPAGPRTLCNACGLKWANKGVLTGGPK---VSNIGMQDPSAKGIEQ--------------------G------DGEAKDSVAITMG-------------------------------------A---NIAPSPNGDNSTMTVDRNL--------------------------------------------------------------------------------------------------------------------------------

>VvZML1

---------------------------------------------------------------------------------------MYGSEAMNLRTQ-----------IEDDDEDDDVAGAEESID---------------------------------------------------------NPQARFEGH------------------VIEDRAVVV------MNGVQDVHHNHLYVPGSDFAPV-----------------------------------------------------------------AGGGGGGGGVDQLTLSFQGEVYVFDAVSPEK------------------------VQAVLL-----L--LGGY-EVP-------------------------TGIPAPGM--------------------------------VPPNQR----GLADFTG---------------------RSSQP----------------------------------------QRAASLSRFREKRKERCFDKK-IRYTVRKEVAL-------------RMQRKKG--------------------------------------------------------------------QFTSSKA-SSDEVGGGASSDWNAAHGSGQ----DEPEILCTHCGTSSKTTPMMRRGPAGPRSLCNACGLKWANKGVLRDLSR---VSS-GVQETSLKATQS--------------------N-------GDANESGAITTV-------------------------------------P---DIV-SSNGDNSAVTAER----------------------------------------------------------------------------------------------------------------------------------

>PpZML4

------------------------------------------------------------------------MDDICGSD-------EPMHMNDNLHLQ--YM-------QDHEHHHGLDQISNVDGVAD----------------------------------------------------------------------------------DHENGNGGA------ELVQADVPSDPMNLSDTRDGMM--------------------------------------------------------------------DHGPENGDQLTLSFQGQVFVFDSVSPEK------------------------VQAVLL-----L--LGGR-EVP-------------------------PSMPAVPV-------------------------------TTQHNNQ----GLTSTPQ---------------------RLSVP----------------------------------------QRLASLIRFREKRKERNFDKK-IRYTVRKEVALRYGICICHNNQIMRMQRKKG--------------------------------------------------------------------QFTSSKP-NNDDSASAVTSSGSNESWSQDGNGSQHQEAVCRHCGINEKCTPMMRRGPDGPRTLCNACGLMWANKGTLRDLSKAAAAAAPQAGQNPSVSKNEDIKPDL------------------------------------------------------------------------------------------------------------------------------------------------------------------------------------------------------------------------------

>VvZML2

--------------------------------------------------------------------MDGIHGNNAGIH-------RPDGQHHPIHVQ--HY-------MQEHDHGMHHMSNGGSMD------------------------------------------------------------------------------------EDHDEGGSG------EGMEGDVQADPGNLADSRGALT------------------------------------------------------------------VQPGGSDNQNQLTLSFQGQVYVFDSVSPEK------------------------VQAVLL-----L--LGGR-EVP-------------------------PTMPALSI---------------------------------AGHNR----ELPGTPQ---------------------RYNVP----------------------------------------HRLASLIRFREKRKERNFDKK-IRYTVRKEVAL-------------RMQRNKG--------------------------------------------------------------------QFTSSKSNHDDSASTTPGWESSWGMAGNG---PINQEIVCRHCGISEKSTPMMRRGPEGPRTLCNACGLMWANKGTLRDLSKAAPEAGQSPSLNQTGENGNFETDQMVLGMAENVSDQA------------------------------------------------------------------------------------------------------------------------------------------------------------------------------------------------------------------

>PpaZML3

-----------------------------------------------------------MSDGDHLVHAQSLQLHHNL---------HPQSLSHGLGHAQHAM-------REMHVHGHGESEGHGQAR-------------------------------------------GDQQVQDHLEVDQVHNHHGHHGHGM----------------HNKEENGVE------VEEHDDDAGDEEGLDEADMHSDGGGNPGD--------------------------------------------------------GPTPLAVRTQGSTQLTLSYQGEVYVFDTVPPKK------------------------VQAVLL-----L--LGGR-EIP-------------------------PGMSGVNM--------------------------------FSHHHK----GLTELPA---------------------RMNMP----------------------------------------QRLASLTRFREKRKERCYDKK-IRYTVRKEVAQ-------------RMQRKKG--------------------------------------------------------------------QFASSRP-TQEEGAPVANWDGTLAPGQTV-APGVHPEVTCVHCGIGERSTPMMRRGPAGPRTLCNACGLMWANKGVLRDLSKNLSITAGGQQQLMLHPQQI-LQQQQHQVAGLQGSVDNAPQ------GVDVQ-GSQQREG-------------------------------------DVQKDMASIVTADGGPVLAAG----------------------------------------------------------------------------------------------------------------------------------

>PpaZML4

-------------------------------------------------------------------MHVHGHGEADGH--------GHVRVDQRVQGH-----------LEGDHAHGNHGHHGHGM-------------------------------------------------------------------------------------HNNEENEAE------VEDHDDDAADEEGLDEANMHSDGGGNPND--------------------------------------------------------GPAPLTVRTQSSTQLTLSYQGEVYVFDAVPPEKV-----------------------VQAVLL-----L--LGGR-EIP-------------------------PGMSGVNI------------------------------SNNHHHHK----GLTDLPA---------------------RMNMP----------------------------------------QRLASLTRFREKRKERCYDKK-IRYTVRKEVAQ-------------RMQRKKG--------------------------------------------------------------------QFASSRP-SQEEGAPVANWDGTQALGQPVGAGGVQPEVICVHCGIGERSTPMMRRGPAGPRTLCNACGLMWANKGVLRDLSKNLSIAPGVQQQLILQSQQVSTFTAL------------------------------------------------------------------------------------------------------------------------------------------------------------------------------------------------------------------------------

>PpaZML2

----------------------------------------------------MRPEEVDVGGMSDVDHLVHGQSLQLHHNI------HQHSLDHALGHAQHGM-------HEMHVHGHGHGEADVQGR-----------------------------------------VDQRVQRHLEGDHVHGHNGHHGHGHGM----------------HHNEENGAG------VEDHDDDAGDEEGLDEAEMHSDGGGNPGD--------------------------------------------------------APPALAIRTQGSTQLTLSYQGEVYVFDAVPPEK------------------------VQAVLL-----L--LGGR-EIP-------------------------PGMSGGNV-------------------------------SSHHHHK----GMPELPS---------------------RMNMP----------------------------------------QRLASLTRFREKRKERCYDKK-IRYTVRKEVAQ-------------RMQRKKG--------------------------------------------------------------------QFASSRP-TQEEGSPVSNWDGTQASGQPL-GPGVQPEVSCVHCGIGERSTPMMRRGPAGPRTLCNACGLMWANKGVLRDLSKNLPMTAGGQQQLMLHPQQIFLQQQQDQVGGLQHSLENVSQ------VADVQMGIQQREV-------------------------------------DEQKSMASMVAVGGGPVVAAG----------------------------------------------------------------------------------------------------------------------------------

>PpZML3

---------------------------------------------------------------------------------------MAAVNPQPLQAR------------PFEEHGRGPIPIEDDEA------------------------------------------------------------------------------------EYEDGGDDG------MEDMEEVHVNSVSVAERGGVGGGGG---------------------------------------------------------------GGVVMASRTSELTLSFEGEVYVFPAVTPEK------------------------VQAVLL-----L--LGGR-DVP-------------------------TGVPTVEV-------------------------------SYDQNTR----GVADTPK---------------------RSNLS----------------------------------------RRIASLVRFREKRKERCFDKK-IRYTVRKEVAQ-------------RMLRKNG--------------------------------------------------------------------QFASLKQ-N----SGDSGWDSAQSGLQDGTSRPETVLRRCQHCGVSENNTPAMRRGPAGPRTLCNACGLMWANKGTLRDLSKGGRNLTMDHIEPGTPIEVKPLLVEGE-FSGNQDEHGTLEGSSKTVIERSNDASVNLDEQ------------DLHETAE-DLTNSLPMGIVSSAN--DEQEPLVELTNPSDTDLEIPTNFD-------------------------------------------------------------------------------------------------------------------------------

>VvZML4

---------------------------------------------------------------------------------------MAMANPQPLQAR--PF-------EDHMQIPIQIHDDDADFE------------------------------------------------------------------------------------VDGGASAPA------DDAMDDVDDDPHINSINPIDHA-------------------------------------------------------------------GVVVASRTSELTLAFEGEVYVFPAVTPEK------------------------VQAVLL-----L--LGGR-DIP-------------------------TGVPTIEV-------------------------------PFDQSNR----GVGDLPK---------------------RSNLS----------------------------------------RRIASLVRFREKRKERCFDKK-IRYTVRKEVAQ-------------RMHRKNG--------------------------------------------------------------------QFASLKE-S----SGPSNWDSAQSLQGDTTPRPETVLRRCQHCGVSENSTPAMRRGPAGPRTLCNACGLMWANKGTLRDLSKGGRNLSLDQIELGTPIDVKPSIMEGENFSGSQDEHITPEDPSKAVAERTDNPSVNADEEVMSSGVYPSSFTDLHETAD-DLSHTLPMGIVHSSGNLDDQETLVELANASETEMDIPGNFD-------------------------------------------------------------------------------------------------------------------------------

>PpZML2

---------------------------------------------------------------------------------------MEMVNAQLLQAR-----------AYEGEDQLVHVPAELEGD------------------------------------------------------------------------------------SDEGGGAKA------AMNGGDQTRRSSGVTMSRCNSA----------------------------------------------------------------------LPSRTSELTIAFEGEVHVFPAVTPDK------------------------VQAVLL-----L--LGGR-DIS-------------------------SSFPSSES-------------------------------LLESNSG----GIGDISR---------------------NSKLS----------------------------------------RRTASLVRFREKRKERCFEKK-IRYTCRKEVAQ-------------RMYRKNG--------------------------------------------------------------------QFASLKD--------DSKIASGNCDSSDGTSCPESVLRRCQHCGISEKSTPAMRRGPAGPRSLCNACGLMWANKGTLRDLTKAGRPIHFDQTELETAADFKPLMLKPENAHLDPDEEGSPEE-SKPIALDTENPPLRLGDE------------DMLETAEAATTNHISIQMENSTVNFDEQENLDEFCNASGTEFEIPANFDEQVVDFYDCNIETHWPGT-------------------------------------------------------------------------------------------------------------

>AtPPD1

-----------------------------------------------------------MDVGVSPAKSILAKPLKLLTEE------DISQLTREDCRK--FL-------KDK---GMRRPSWNKSQA------------------------------IQQVLSLKALYEPGDDSGAGIFRKILVSQPVNPPRVTT----------------TLIEPSNEL------EACGRVSYPEDNGACHRMDSPRSAEFSGGSGHFVSEKDGHKT-----------------------------------------TISPRSPAETSELVGQMTIFYSGKVNVYDGIPPEK------------------------ARSIMH-----F---AAN-PID-------------------------LPENGIFA--------------------------SSRMISKLISKE----KMMELPQKGLEKAN--------------SSRDS----------------------------------GMEGQANRKVSLQRYREKRKDRKFSKA-KKCPGVASSSLE---------------------------------------------------------------------------------------MFLNCQPRMKAAYSQNLGCTGSPLHSQSPESQTKSPNLSVDLNSEGI------------------------------------------------------------------------------------------------------------------------------------------------------------------------------------------------------------------------------------------------------------------------------------------

>AtPPD2

-----------------------------------------------------------MDVGVTTAKSILEKPLKLLTEE------DISQLTREDCRK--FL-------KEK---GMRRPSWNKSQA------------------------------IQQVLSLKALYEPGDDSGAGILRKILVSQPPNPPRVTT----------------TLIEPRNEL------EACGRIPLQEDDGACHRRDSPRSAEFSGSSGQFVADKDSHKTV----------------------------------------SVSPRSPAETNAVVGQMTIFYSGKVNVYDGVPPEK------------------------ARSIMH-----F---AAN-PID-------------------------LPENGIFA--------------------------SSRMISKPMSKE----KMVELPQYGLEKAP--------------ASRDS----------------------------------DVEGQANRKVSLQRYLEKRKDRRFSKT-KKAPGVASSSLE---------------------------------------------------------------------------------------MFLNRQPRMNAAYSQNLSGTGHCESPENQTKSPNISVDLNSDLNSEDN-----------------------------------------------------------------------------------------------------------------------------------------------------------------------------------------------------------------------------------------------------------------------------------------

>MdPPD2

-----------------------------------------------------------MNAATTTFPSILXKPLNQLTED------DISQLTREDCRK--YL----------KEKGMRRPSWNKSQA------------------------------IQQVISLKALLEPNEDSGAGALRKIVVSAQTTTATTQ-----------------RAASNSADS------AKEASADVQASVSADEPATHPRNERPKSVPEDPPVDADTAAI-------------------------------------------SPRNQCTTDALVRQMTIFYSGKVNVYDGVPPDKVNEAFYLNGDLEISLPMQRYMDLQARAILH-----F---AAG-----------------------------PNHLLLDNQFGGAAAERSLXCQYQTAGDKDGPFPPSATISQSMQTG----KFGEYTQQYWEKGN--------------STRDP----------------------------------DAEGQASRKVSLQRYREKRKD-RERLK-IKKNSGANSSL-------------EVYL-----------------------------------------------------------------------NHQLRTHTSNGNSSQSGTSSPPQPGLLQ--TAENQPKIRCLPVDLNEKDILERQA----------------------------------------------------------------------------------------------------------------------------------------------------------------------------------------------------------------------------------------------------------------------------------

>PpPPD1

MRRRRAYSLSLSLSLSHSLLCLLPFPLFPPIPLSPSQLPRNYQPAQIAFAGLSVAETPAMNATTTSFRSILEKPLNQLTED------DISQLTREDCRK--YL----------KEKGMRRPSWNKSQA------------------------------IQQVISLKALLEPNDDTGAGALRRIVVSPHTTTPRAASNSAG------------SAKEASADV------QVSVSADEPVPYQKPVQEDRPADADTKAI--------------------------------------------------------SPRNQCTTDASVRQMTIFYCGKVNVYDGVPPDK------------------------ARAIMH-----L---AAR-----------------------------PNHLPLDNQFGGTAALRSLRCQFQTAGDKDGFLPPSATFSQAMQTE----KIGEYTQQYWEKGN--------------STRDP----------------------------------DAEGQASRKVSLERYREKRKD-RGRLK-IKKNIGSSSSL---------------EVFLN--------------------------------------------------------------------HQLRTHT-SNGNSSQSGTSSPPQPGLLQ--TADNQPKSLCLPVDLNDKDILERRT----------------------------------------------------------------------------------------------------------------------------------------------------------------------------------------------------------------------------------------------------------------------------------

>MdPPD1

-----------------------------------------------------------MNAPTTTFRSILEKPLNQLTED------DISQLTREDCRK--YL-------KEKGDPAGYFAQGAAGAQ-------------------------RRFRRRSSQKDCRFASYDHRHHPARQYFRMCLFPEKXKTGEKI----------------TAASNSADS------AKEVSPDVQASVSADELAPHPRNEPPKPAPEDPPVYADTTAISLRLIFSPWVMAYAWVDYIRIKGLTEVTLRLGCIFSVFLKTGMKPKNHCTTDASVSKMTIFYSGKVNVYDGVPPDKVNEAFSLNGDXEISLPMQGYMDLQARAILH-----L---AAG-----------------------------PNHLLLDNQFGGAAAARSLHCQFQTAGDKDGLFLPSATISQAMQTGNFTEKVXEYTQQYWEKGN--------------NTRDP----------------------------------DAEGQANRKVSLQRYREKRKD-REKLK-IKKNIGSNTSL-------------EVYLNRQL-------------------------------------------------------------------RXHTSNGNSSQYGTSSPPQPELLQTAENQPRFRCLPVDLNEKGRLDAEFCYVMKNRGRGSVGASEKCITFRLTLQFLGFHFKEPLWAELQSSFL-------------------------------------------------------------------------------------------------------------------------------------------------------------------------------------------------------------------------------------------

>VvPPD1

-----------------------------------------------------------MNPGVTTLRSILDKPLHELTEE------DISQLTREDCRK--YL----------KEKGMRRPSWNKSQA------------------------------IQQVISLKSLLETSEGSGAGVLRKITDSPPAENLPPVTSNSADSGKELSADIQ-ISVSADELV------PLPPKDHHPESTPSGELASRPPEADTKHT--------------------------------------------------------CPRSPGATNCLVGQMTIFYCGKVNVYDGVPDDK------------------------AQAIMH-----L---AAS-----------------------------PFHLPSDDPFSGAAMLCSSPCHLHTANVKHGHIPPRAMVSQTMQTE----KFTEYSQQYREEVNFTRGHGSEALSGLRTVGSPTARPTEDMEQTTCLTIWGTFRYKVMPFEIYEGIMDVEGQVDRKLSLQRYFEKRKD-RFKSR-KKIGLPSGSLE--------------MYV-----------------------------------------------------------------------NHQARTQPSNGQSSRSGTSSPPQHGLSHTLCSSADNHTKNFTPFVDLNSKDIQES----------------------------------------------------------------------------------------------------------------------------------------------------------------------------------------------------------------------------------------------------------------------------------

>VvPPD2

-----------------------------------------------------------MSPENANIRSLLDKPLHQLTED------DISQLTREDCRK--YL-------KEK---GMRRPSWNKSQA--------------------------IQQVISLKTLLETTSDCGGGDAAGARKKLFVPPPENQHRVPLT---------------RISVSDEES------VPYQRQDPPKPDISGDTEAHLLAAADSDS-------------------------------------------------------IPPRTLDAMNGPAGQMTIFYCGKVNVYDDVSMDK------------------------AKAIMQ-----L---AAS-----------------------------SLHLHQEAPCDGTPELLPFSCHLRAASVKIGPSSPTVIYPTLQTVK----MTENCQLHREESNIFR------------EDNHP----------------------------------AAEVPTSRKASVQRYLEKRKD-RFKSK-KRGGMPSSAGL-------------DIYL-----------------------------------------------------------------------NHRVGDQIPNDQSNQSDACSLSHCRAHHIPTPCSLVENMTKHTNLSADLNIKDVQEH--------------------------------------------------------------------------------------------------------------------------------------------------------------------------------------------------------------------------------------------------------------------------------

>MdML1

-------------------------------------------------MSLPLSHAAKGXSPTLPPIFLLDFLRXPRVSRFLLLFFSPAKGPCLPWILLFCLSXATTLNGAREASASFFVCWALNPT----------------------QIQTCGAHASPSIAAWANFLANFSATHDGTWLSKLPLCGYAWGYPRNG--------------RIRSPEFNV------QHRGGVTQSNQVDDQDDDVEEPIDNXNIRFEDSTAIPPNQLYL---------------------------------------PSSEYPPPPAANGASDQLTLSFQGEVYVFDAVSPDK------------------------VQAVLL-----L---LGGYEIP-------------------------SGIPSMGP--------------------------------VPLNQQ----GMNDLPA---------------------KPIQP----------------------------------------QRAASLSRFREKRKERCFDKK-IRYTVRKEVAL----------------------------------------------------------------------------------------RYGKIITYLIIFFNFS-------------------------------------------------------------------------------------------------------------------------------------------------------------------------------------------------------------------------------------------------------------------------------------------------------------------------

>OsJAZ15

----------------------------------------------------------MDAVGAAGGGAMLPAAARRG---------QPPQPPCMTTAP------------EQQAAAGGAVIWPAAAA------------------------------------------------------------------------------------AEAKEKMVV------DARTMQLFPTRSADGVVVSPAPAPAAAQERRRPEV--------------------------------------------------HVTPSVPATAPTAPLTIVYGGQVLVFEHYTAEA------------------------AEKLVQRTQHLLAAAAGG-GGG-------------------------NKNNNVTV--------------------VTPPPDEPPMLLPPPQMP----AASGVSA------------------------------------------------------------GGVMPIARKASLQRFLQKRKQ-K-------------------------------------------------------------------------------------------------------------------------------------------------------------------------------------------------------------------------------------------------------------------------------------------------------------------------------------------------------------------------------------------------------------------------------------------------
